# Methods for copy number aberration detection from single-cell DNA-sequencing data

**DOI:** 10.1186/s13059-020-02119-8

**Published:** 2020-08-17

**Authors:** Xian F. Mallory, Mohammadamin Edrisi, Nicholas Navin, Luay Nakhleh

**Affiliations:** 1 Department of Computer Science, Rice University, Houston, TX USA; 2 Department of Computer Science, Florida State University, Tallahassee, FL USA; 3 Department of Genetics, the University of Texas M.D. Anderson Cancer Center, Houston, TX USA

**Keywords:** Tumor evolution, Intra-tumor heterogeneity, Single-cell DNA sequencing, Copy number aberrations

## Abstract

Copy number aberrations (CNAs), which are pathogenic copy number variations (CNVs), play an important role in the initiation and progression of cancer. Single-cell DNA-sequencing (scDNAseq) technologies produce data that is ideal for inferring CNAs. In this review, we review eight methods that have been developed for detecting CNAs in scDNAseq data, and categorize them according to the steps of a seven-step pipeline that they employ. Furthermore, we review models and methods for evolutionary analyses of CNAs from scDNAseq data and highlight advances and future research directions for computational methods for CNA detection from scDNAseq data.

## Background

Intra-tumor heterogeneity (ITH) has been a major confounding factor in cancer prognosis, treatment, and prevention [1–4]. ITH describes the phenomenon in cancer when one tumor contains multiple subclones, each characterized by a certain group of mutations [2]. When ITH is not fully characterized, cancer treatment tends to target only major clones whereas the small subclones may grow mid- or post-treatment, leading to cancer relapse [5–7]. Fully characterizing ITH helps understanding cancer growth and thus can improve cancer treatment and prevention [1, 8–13]. To achieve that, one needs to correctly detect mutations in each cell and infer the phylogenetic tree of the subclones.

Single-cell DNA-sequencing (scDNAseq) data make it possible to read the DNA one cell at a time and identify the individual mutations that occurred. To achieve this goal, computational tools must be developed for detecting mutations in the individual cells and inferring the cell-lineage tree that describes the evolutionary history of the cells from their most recent common ancestral cell, along with the mutations that occurred. While mutations can first be called for the individual sequenced cells and then a tree is built from those, it is expected that carrying out both tasks simultaneously would be more powerful in producing more accurate inferences, as the tree structure captures the temporal dependencies across cells and can serve as a “regularizer” on the number of inferred mutational events [14, 15].

Two types of mutations have been the focus of detection from scDNAseq data: single nucleotide variants (SNVs) and copy number aberrations (CNAs). While there have been quite a few methods specifically developed for detecting SNVs from scDNAseq data, there are only a limited number of CNA detection methods for such data. Moreover, quite a few methods that have been applied to detect CNAs from scDNAseq data were originally designed for other types of data, including array-CGH [16] and bulk next-generation sequencing data. Degenerate oligonucleotide-primed PCR (DOP-PCR) [17–20] and Transposon Barcoded library (TnBC) [21] are two single-cell DNA amplification protocols generating data suitable for CNA detection and are limited in terms of their coverage depth and uniformity. It is therefore important to study the existing methods in terms of how well they can be adapted to scDNAseq data. Additionally, since aneuploidy can be commonly seen in tumor [22], it is essential to discuss the adaptability of the methods to aneuploid data, as diploidy is assumed by many existing CNA detection methods. Finally, while each SNV occurs at a single nucleotide, CNAs could affect a whole chromosome (chromosomal duplication and loss) or a focal region (less than 98% of a chromosome arm [23]). For CNAs, the chromosomal events are a result of genomic instability due to segregation error during anaphase [24], whereas most of the focal ones are a result of the gain of selective advantage during tumor progression [23, 25, 26], although it is not unlikely that a focal CNA becomes abundant just by chance. As demonstrated in [23], CNAs of different sizes vary in their abundances and rates.

Inferring a phylogenetic tree based on CNAs detected from scDNAseq data to capture the cell-lineage tree which is crucial for unraveling ITH yet has not been extensively studied or pursued [27]. Much work has been done on inferring such trees from SNV scDNAseq data [28–33]. In all these methods, SNVs are assumed to be independent, which greatly simplifies the modeling and inference task. However, CNAs span genomic intervals and can overlap each other during evolution rendering the independence assumption among neighboring loci potentially misleading. This issue notwithstanding, the independence assumption is for the most part employed by existing methods in order to make the inference task computationally tractable.

Finally, as more studies are published using inferred CNAs and phylogenetic trees estimated thereon, it is important to understand how to validate the inference results in the absence of a ground truth.

In this paper, we review eight methods for detecting CNV/CNAs from the perspective of the application to scDNAseq data of human cancer genomes. We discuss these methods in terms of seven general steps that are often applied to scDNAseq data for CNA detection, and highlight the strengths and limitations of each method. A major step in CNA detection is *segmentation*, which amounts to partitioning a genome into segments so that the genomic region that corresponds to a segment has a single copy number and every two adjacent segments differ in their copy numbers. We summarize three general approaches for this step which are based on a sliding window, an objective function, or hidden Markov models (HMMs). For each method, we categorize it based on the seven steps and into the three segmentation approaches, as well as some other features that are required in scDNAseq data. We end the review with a discussion of evolutionary analysis of CNAs, focusing mainly on techniques from phylogenetics and molecular evolution that could be relevant in this context.

## Particularities of scDNAseq

scDNAseq is limited by its DNA quantity: a single cell’s genomic DNA quantity is only 6 pg, not enough for massively parallel whole-genome sequencing [34, 35]. Whole-genome amplification (WGA) technologies in DNA library preparation, e.g., multiple displacement amplification (MDA) [36–38], DOP-PCR [17–19], and multiple annealing and looping-based amplification cycles (MALBAC) [39], can alleviate this issue. However, the uniformity of the coverage diminishes due to the amplification bias [40]. The analysis of a non-uniformly sequenced genome tends to contain false-positive CNA calls as it is computationally challenging to distinguish whether read-count fluctuations are due to amplification biases or to true CNAs [41]. Therefore, it is important to measure the non-uniformity of genomic coverage before CNA calling, for example, by measuring the variance of the read counts or the Gini index (a summary measure of the fluctuation of the read counts across bins) for a cell. In addition to genomic uniformity, other scDNAseq specifications that also concern CNA calling are the depth of coverage and the throughput. Depth of coverage defines the size range and the resolution of the CNAs being called. The higher the coverage, the smaller the lower bound of the size of CNAs that can be detected, and the higher the resolution of the CNA boundaries. For single cells sequenced at 0.02–0.1 × coverage, the CNA sizes range from megabases to chromosomal length [42], and the resolution is as low as hundreds of kilobases [18, 42], if not on the megabase level [43]. The throughput of scDNAseq relates how many cells can be sequenced simultaneously and how fast the turnaround time is. Higher throughput allows scalability of scDNAseq and thus facilitates recovering the tumor history from the same sample. Also, typically, methods that have a higher throughput tend to have a lower cost [44] due to the smaller requirement of labor and thus can afford the sequencing of thousands of cells for one sample. Table 1 lists the specifications of the existing scDNAseq technologies in terms of the uniformity, depth of coverage, and throughput, as well as suitability for CNA calling. While we refer the readers to [45–47] for more detailed information, we would like to stress that of all technologies, only Direct Library Preparation (DLP) [40] performs single-cell library preparation without pre-amplification. However, despite its advantages of low cost, efficient labor, and sequencing effort, the Lorenz curve (which captures the unevenness of the coverage) shown in [40] demonstrates that its coverage fluctuation is larger than other technologies such as DOP-PCR for CNA calling at the single-cell level. As expected, when multiple cells are combined together, the coverage fluctuation improves [40]. But such improvement is contingent upon the prior knowledge of the ITH in the cell population, which is rather one of the objectives of the scDNAseq studies.

**Table 1:** Peculiarities of scDNAseq technologies

Method	Uniformity	Coverage	Throughput	Suitability for CNA
MDA [36–38]	Low	High	Low	N
DOP-PCR [17–19]	High	Low	High	Y
MALBAC [39]	High	High	Low	Y
C-PCR-L [44]	High	Low	High	Y
SCI-seq (xSDS) [48]	Medium	Low	High	Y^1^
DLP [40]	High	Low	High	Y
LIANTI [49]	High	High	Low^2^	Y
TnBC [21]	High	Low	High	Y
Flow cytometry [50, 51]	High	Low	High	Y
10x [43]	High	Low	High	Y
DLP+ [52]	High	Low	High	Y

Eleven scDNAseq technologies are listed. Uniformity, coverage, throughput, and suitability for CNA refer to the uniformity of sequencing coverage, the sequencing coverage over the whole genome, the number of cells that can be sequenced at one time, and whether the technology is suitable for CNA calling

^1^With cell filtering

^2^High-throughput sequencing can be achieved by adding combinatorial cellular barcodes

All technologies listed in Table 1 are suitable for whole-genome sequencing. For targeted sequencing, Mission Bio’s Tapestri platform can sequence 2k to 10k cells each at 40 ×–80 × coverage at a magnitude of lower sequencing cost [53], facilitating the detection of single nucleotide variations (SNV) and CNAs simultaneously from the same cell. However, since this technology is targeted at a small set of genomic regions, major CNAs can be missed.

In addition to low and uneven coverage—whose degree can depend on the sequencing technology [54]—scDNAseq data also suffers from a high allelic dropout (ADO) rate, doublets (where two or more cells are accidentally mixed for sequencing), and a high rate of false C >T calls [55]. The C >T bias, though being a major confounding issue in SNV calling, does not affect CNA calling due to the megabase size and low resolution of CNAs that are of interest. The same applies to ADO, as most of the existing CNA calling methods do not distinguish major and minor alleles (for a given SNV, the most common and less frequent alleles are called major and minor alleles, respectively). To the best of our knowledge, the only CNA detection method that is allele-specific is CHISEL [56], which we review below. Generally speaking, since CNAs occur at individual alleles, allele-specific CNA detectors have an advantage of inferring the evolutionary history of the cells. However, resolving allele-specific CNAs requires phasing, which is to distinguish CNAs based on the alleles at which they occur and group them by the alleles, a step that, for example, is accomplished by using SNVs in CHISEL [56]. However, scDNAseq’s low coverage makes it very challenging to do phasing based on SNVs. In addition to low and uneven coverage, errors due to doublets could be problematic for CNA detection. A doublet refers to two single cells being deposited into the same well and taken as one cell in the following computational analysis [20]. When the two cells come from two clones, the resulting CNA profile is a mixture of the two, potentially resulting in non-integer underlying copy numbers, thus violating an assumption of most CNA calling methods. The doublet rate can be as high as 10% [20], but the effect of doublets on the detection of breakpoints is less than their effect on the inference of absolute copy numbers, since the breakpoint signal is still present in the doublet even though such signal may be diluted by the other cell not carrying the same breakpoint. However, cell contamination during cell extraction and pre-amplification process may lead to non-integer copy numbers and false-positive CNAs [57] even to a larger extent as the contaminant cells, by definition, are not from the same sample.

## General steps of scDNAseq CNA detection pipelines

**Fig. 1:**
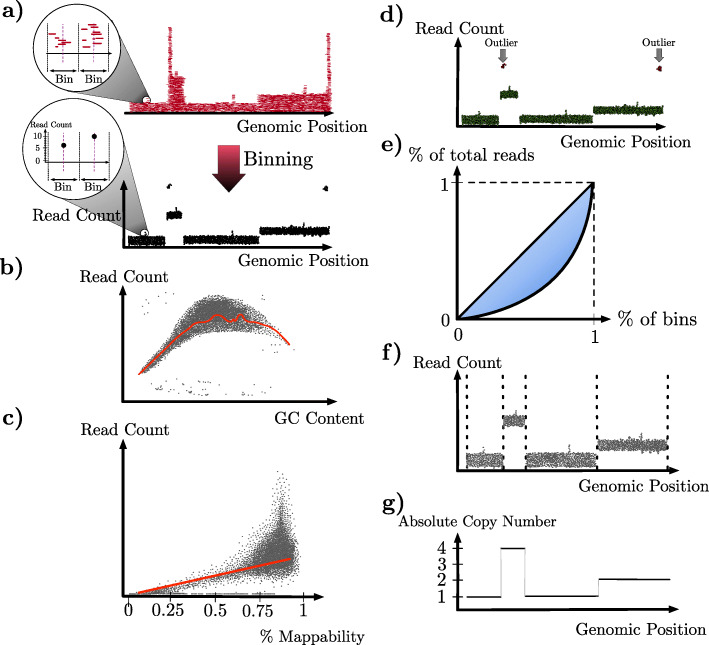
The seven steps in CNA detection in single-cell sequencing. **a** Binning. The number of reads within each bin (bottom) is computed from the pileup of the reads according to where they align (top). **b** GC correction. Scatter plot of read count per bin with respect to the GC content of the bin. The red curve represents the corresponding regression. **c** Mappability correction. Scatter plot of read count per bin with respect to the mappability of the bin. The red curve represents the corresponding regression. **d** Removal of outlier bins. Scatter plot of read count per bin with respect to the genomic position is shown. Outlier bins are shown in red, in contrast with the rest of the genome which are in green. **e** Removal of outlier cells. A Lorenz curve for the read count at all bins is shown. Gini coefficient is twice the highlighted area between the Lorenz curve and the diagonal line. The higher the Gini coefficient, the more likely the cell is an outlier. **f** Segmentation. Scatter plot of read count per bin with respect to the genomic position is shown. Dotted vertical lines correspond to the segments’ boundaries. **g** Calling the absolute copy numbers. The copy number—a non-negative integer—for each segment is determined

In the absence of copy number aberrations and/or variations, a diploid genome has precisely two copies of each chromosome. However, when CNAs occur, the number of copies varies across the genome. In this case, *segmentation* is the task of partitioning the genome into maximum-length contiguous regions such that the copy number at each site within a region is the same. Each such region is called a *segment*, and the copy number shared by all the sites within that segment is referred to as the *absolute copy number*. CNA detection refers to the task of identifying the segments and absolute copy numbers across a given genome. In the case of a diploid genome with no CNAs, there is one segment, which is the whole genome, and the absolute copy number is 2. In general, a method for CNA detection from single-cell DNA data follows some or all of seven steps, illustrated in Fig. 1, five of which are aimed at data wrangling, and two that are aimed at the main tasks of CNA detection, namely segmentation and calling absolute copy numbers.

### scDNAseq data wrangling prior to CNA detection

scDNAseq data wrangling prior to CNA detection most often involves the following five steps:

1. *Binning.* Partitioning the genome into fixed- or variable-size bins so that the resolution at which the genome is segmented and copy numbers are called is defined by bins rather than by individual sites.
2. *GC correction.* Correcting read counts according to the GC content in the corresponding genomic region to remove the GC content’s effect on the read counts.
3. *Mappability correction.* Correcting read counts according to how mappable a genomic region is. The higher the repetitiveness in a region, the lower its mappability, and the lower the number of uniquely aligned reads.
4. *Removal of outlier bins.* Identifying and excluding bins that have extremely high read count regardless of the actual copy number in such bins. These bins often occur near the centromere and telomere of each chromosome.
5. *Removal of outlier cells.* Identifying and excluding cells whose read-count profiles are low in signal-to-noise ratio (SNR). Such cells either have noisy read counts or are low in sequence coverage.

We now briefly describe each of the five steps.

#### Binning

Bins are computed so that reads within a bin are aggregated to reduce the effects of variable amplification and sequence sampling [58]. Most methods that employ binning use bins of a fixed size (all bins have the same size). However, the use of variable-size bins has been proposed [18] to avoid false-positive deletion calls (i.e., false calling of breakpoints) in repetitive regions, as a result of the removal of low mapping quality reads. Bins of variable sizes are determined as follows. First, the reference genome is sequenced in silico to produce a set of reads. The reads are then aligned back to the reference, and those that cannot be uniquely aligned are removed. The genome is then divided into variable-size bins, each bin containing roughly the same number of uniquely aligned reads. For the repetitive regions, their variable bin size is expected to be larger since the number of uniquely aligned reads is smaller [18].

While using variable-size bins effectively reduces false-positive deletion calls, it is restricted in a few aspects. First, the bin size is with respect to a specific reference genome. Different reference genomes could give rise to different binning outcomes. Second, the bin size is affected by the particular read length being simulated. More generally, determining the optimal bin size (variable or fixed) is hard due to variable read coverage in single-cell data. As mentioned in [57], there needs to be a certain number of reads in each bin so that the resulting read counts follow a normal distribution, according to the central limit theorem. Furthermore, the use of bins could limit the segmentation resolution since bins restrict the segment boundaries to be a subset of the bin boundaries. Finally, the use of bins may generate false-negative calls (i.e., missing breakpoints) for CNAs whose size is close to or smaller than the bin size as the number of the bins in that segment is so small that the signal is often mistaken as noise and will not be called as a CNA. For scDNAseq, this could lead to false-negative CNAs, which are hundreds of thousands of base pairs long, as the bin size is often around 200 kbp [27].

#### GC correction

GC correction is a necessary step due to the dropping of read coverage at the regions with extreme GC contents [59, 60], a phenomenon referred to as GC bias. As the GC content is the percentage of the nucleotides being G or C often measured within a certain genomic region, a binning step is needed to divide the whole genome into multiple bins. Thus, a binning step always precedes a GC correction step for most of the methods. The GC correction starts with modeling the change of read count with respect to GC content, and it is specific to each cell, as different sample preparation libraries may produce different curves of read count with respect to GC content. The read count at each bin is then normalized and corrected. Most methods correct GC bias under the assumption that a majority of the genome is diploid and does not harbor copy number variations/alterations. Such an assumption, however, is not necessarily true. Specifically, tumor genomes can be aneuploid and thus contain chromosomal amplifications [27]. When no copy number dominates a genome’s copy number profile, one has to consider which copy number this bin is at when modeling its read count by the GC content [61]. To do so, a pre-segmentation and estimation of the absolute copy number is required for GC correction. SCOPE [62] provides a cell- and bin-specific GC correction that addresses such a scenario. GC correction strategy in single-cell data does not differ much from that of bulk sequencing data, though the true copy number in single-cell data is an integer, whereas it needs not be an integer in bulk sequencing data as it is an average of copy numbers from thousands of cells.

#### Mappability correction

Mappability of a genomic region is determined by the percentage of the uniquely mappable positions in this region. Similar to GC correction, a binning step is required so that the whole genome can be divided into multiple bins for a measure of the mappability of each bin. If a bin is mainly composed of N nucleotides or repetitive regions, its mappability is possibly low. The process of determining the uniquely mappable positions is described above. Correction of the read counts according to mappability can follow the same process as GC correction, i.e., learning the curve of the read count with respect to the mappability of each bin, and normalizing the read count accordingly to remove the effect of the mappability by LOWESS regression or a parametric function. As described above, the use of variable-size bins aims to correct the uneven mappability, resulting in the same number of uniquely mappable reads in each bin. However, correcting mappability based on a pre-computed mappability file constrains the CNA detection tool to certain human genome reference versions.

#### Removal of outlier bins

Bins that are identified as outliers are removed from further analysis by their genomic positions, GC content, or their read counts. Most often, bins at centromere or telomere of a chromosome, with extreme GC content or with zero or extremely high read counts, are identified as outliers. Removing outlier bins can reduce false-positive calls. But how to select outlier bins makes a difference among methods. The selection process is more flexible and adaptable to the data if it is data-driven, i.e., the threshold of determining which bins are outliers is a function of the data instead of given directly by the CNA detection tools. Ginkgo [58], for example, blacklists “bad bins” using 54 existing normal cells without input from the data at hand, whereas HMMcopy [16] and SCOPE [62] model the read count in each bin as a distribution and detect the outliers in the data based on this distribution.

#### Removal of outlier cells

Cells are identified as outliers and removed from further analysis if their coverage is lower than expected [27, 57], or their Gini coefficient is larger than expected [62]. The higher the Gini coefficient, the more heterogeneity/fluctuation in the read depth, and the harder to infer CNAs. Both low coverage and large Gini coefficient indicate potential failed library preparation. Removing outlier cells is important for downstream analyses such as recovering the phylogenetic tree or clustering the cells. Cells with extremely high Gini coefficient are expected to have a large number of false-positive CNA calls, as the read counts become noisy and the CNA detection tools may falsely count them as CNA signals. Such cells thus shall not be considered in CNA inference.

### Segmentation and absolute copy number calling

The last two steps of a 7-step CNA detection protocol are as follows:

6 *Segmentation.* Identifying the boundaries (physical locations) between genomic regions that have different absolute copy numbers.
7 *Calling the absolute copy numbers.* Inferring the actual absolute copy number within each segment.

#### Segmentation

**Fig. 2:**
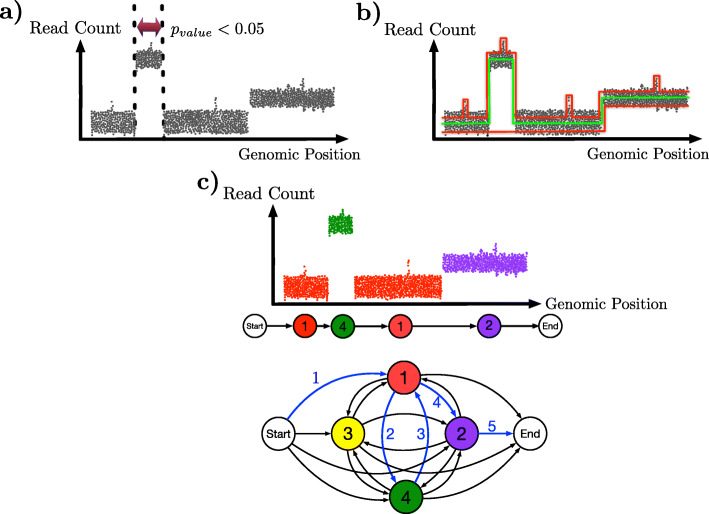
The three approaches for segmentation. In all three panels, a scatter plot of the read count per bin with respect to the genomic position of the bin is shown. **a** The sliding-window approach. A window is passed across the genome, and a genomic region within a window that is significantly different in terms of read count from the rest of the genome (e.g., the window defined by the two dotted vertical lines) is declared as a segment. **b** The objective function-based approach. Three piecewise constant functions are shown (two in red and one in green) and represent segmentation candidates. Each piece in the function corresponds to a segment, and the value of the piece corresponds to the copy number at that segment. The function in green is the optimal one with respect to the fidelity to the data and the constraint on the number of breakpoints, whereas the two in red are either over-segmented (top) or under-segmented (bottom). **c** The HMM-based approach. States of the HMM correspond to the different copy numbers, and a transition between two different states indicates a change in the segment. In the read-count panel, colors of the dots represent the absolute copy number of the various genomic bins (red for 1, yellow for 2, and green for 4) as obtained by parsing the data with respect to the HMM (bottom). The actual path of the state transitions is shown in the middle and highlighted with blue arrows on the HMM as well. The arrows are numbered to indicate the order of the transitions

There are three approaches to segmentation (Fig. 2): a sliding-window approach, an objective function-based approach, and an HMM-based approach. The sliding-window approach segments the genome by statistical testing, looking for regions whose read counts differ greatly from those of the other regions. Methods that use the sliding-window approach do not calculate the absolute copy number simultaneously with segmentation. A post-processing approach is needed, which usually involves testing different candidates of ploidy and selecting the one whose resulting copy number profile is as close to integer numbers as possible. The objective function-based approach combines the approximation to the data and the limitation of the breakpoints in one formula. Such approaches model the (normalized) read count by a piecewise constant function, so that the function (i) is in fidelity to the data as much as possible and (ii) has as few changes as possible. Like the sliding-window approach, methods based on the objective function approach do not necessarily simultaneously assign absolute copy number to each segment either. Finally, in the HMM-based approach, states correspond to the different possible absolute copy numbers, and transitions between states capture the segmentation (i.e., transition out of a state at bin *i* denotes that bins *i*−1 and *i* belong to two different segments). Due to its ability to model hundreds of cells in one objective function, the objective function approach is more suitable for simultaneous breakpoint identification across sampled cells.

#### Calling the absolute copy numbers

Except for HMM-based methods, which identify the absolute copy number along with the segmentation, all other methods require a post-processing step to identify the absolute copy number for each segment after segmentation has been obtained. When DNA ploidy information is available, e.g., through flow cytometry data, the absolute copy number for a segment can be calculated by multiplying the genome-wide ploidy by the read count in that segment, divided by the mean read count across the genome [27, 58]. When DNA ploidy information is not available, a copy number multiplier is to be found which best approximates the normalized read counts in each bin to its closest integer number. Since HMM-based methods produce both a segmentation and absolute copy numbers at the same time, the selection of ploidy is essential in determining the copy number profile genome-wide. We observed that HMMcopy does not always select the right ploidy [54], leading to wrong copy number inference for the entire genome. Visualization of the CNA profiles of a group of cells is usually done by heatmaps, one example of which can be seen in [27].

## Review of CNA detection methods

In this section, we review eight methods for calling CNAs that have been designed for or applied to scDNAseq data. Their characteristics in terms of the aforementioned steps are listed in Table 2. We pay particular attention to and discuss how each method is designed for or adapted to scDNAseq data. We divide the eight methods into two categories based on whether they handle a single sample/cell at a time, or multiple ones at once, as the latter potentially fits scDNAseq data better because it pools the information shared among the single cells.

**Table 2:** Eight methods for calling CNVs or CNAs

**Method**	**Reference**	**Features**				**Seven steps of Fig. **1						
		S	D	N	P	1	2	3	4	5	6	7
HMMcopy	Shah et al. [16]	S	N	N	Y	Y	Y	Y	Y	N	3	Y
Ginkgo	Garvin et al. [58]	S	N	N	N	Y	Y	N	Y	Y	1	Y
AneuFinder	Bakker et al. [50]	S	N	N	Y	Y	Y	N	Y	Y	3	Y
SCNV	Wang et al. [57]	S	N	Y	Y^1^	N	Y	N	N	Y	1	Y
CopyNumber	Nilsen et al. [69]	M	N	N	N	Y	N	N	Y	N	2	N
SCOPE	Wang et al. [62]	M	N	Y	N	Y	Y	Y	Y	Y	1	Y
CHISEL	Zaccaria et al. [56]	M	Y	N	Y	Y	Y	N	Y	N	2	Y
SCICoNE	Kuipers et al. [15]	M	N	N	N	Y	Y	Y	N	N	2	Y

Four features of the methods are highlighted. S, whether the method applies to a single sample (S) or multiple samples simultaneously (M); D, whether the method assumes diploidy of the sample (Y) or not (N); N, whether the method requires genome of a normal cell (Y) or not (N); P, whether the method is parametric (Y) or not (N). Each method is also classified according to which of the seven steps of Fig. 1 it employs. 1, the method applies binning (Y) or not (N); 2, the method applies GC correction (Y) or not (N); 3, the method applies mappability correction (Y) or not (N); 4, the method removes outlier bins (Y) or not (N); 5, the method removes outlier cells (Y) or not (N); 6, the method applies sliding window (1), objective function fitting (2), or HMM (3) for segmentation; and 7, the method calls the absolute copy number (Y) or not (N). “NA” denotes that the step is not applicable to the method

^1^The model is automatically calibrated given the identified normal cells

### Single sample/cell-based methods

#### HMMcopy and AneuFinder

Both HMMcopy [16] and AneuFinder [50, 51] are HMM-based approaches. HMMcopy was originally designed for array CGH data, but has been extensively applied to large-scale scDNAseq data [40, 63, 64]. Both HMMcopy and AneuFinder use an eleven-state HMM, where the states correspond to copy number counts 0,1,…,10. In addition to GC correction which is also a step included in HMMcopy, AneuFinder does quality control using a multivariate clustering approach implemented in mclust [65–67] which combines several metrics defined in [50]. HMMcopy also models the outlier bins to reduce false-positive calls [16].

One advantage of HMMcopy and AneuFinder is their ability to simultaneously infer segmentation and absolute copy numbers. As shown in [54], despite the possibility of selecting the wrong ploidy, HMMcopy achieved high precision and recall (around 0.8 for recall and 0.7 for precision) in both segmentation and copy number inference in a simulation study of single cells. Another advantage of HMMcopy is that its computational burden is relatively light. For 1000 cells at the depth of 0.04 ×, it takes about 7 h to finish all jobs on an Intel(R) CPU E5-2650 v2, 2.60GHz with < 1 Gb memory [54]. The challenges with using HMMcopy include its requiring manual calibration of more than ten parameters, as well as its inability to reliably estimate the ploidy, which leads to incorrect copy number estimation, as demonstrated in [54]. Furthermore, in the same study, it was observed that AneuFinder’s recall dropped dramatically when the ploidy increased, and its precision was overall low for all ploidies (< 0.6). HMMcopy’s segmentation result, however, is relatively robust to the varying ploidies. One shortcoming of HMMcopy is that it uses a mappability file whose kmer size and reference version are restricted to a small pool (the latest reference version is GRCh37).

#### Ginkgo

Ginkgo [58] is a web platform for analyzing single-cell copy number variations, although it can also be run locally. It uses variable bin strategy to segment the genome into bins followed by GC correction. To remove outlier bins, Ginkgo was applied to 54 human normal diploid cells and a list of “bad bins” was created by testing the *p* value of the normalized read count for each bin. It then uses a circular binary segmentation (CBS) [68] approach and identifies the absolute copy number by selecting the optimal multiplier to best align the normalized read count with an integer number, i.e., the copy number. In addition to segmentation and calling the absolute copy number, Ginkgo also clusters the cells, infers the phylogenetic tree of the cells, and provides interactive plots to visualize GC bias, Lorenz curve, the resulting copy number profiles, and phylogenetic tree. In addition, users can search specific genes of interest and select subsets of cells for comparison purpose. The advantages of Ginkgo include its web-based platform, the post-processing steps such as phylogenetic tree inference and clustering of the cells, and its interactive platform for visualizing the data. The limitations of Ginkgo include the fact that the list of bad bins is learned from a fixed set of normal cells instead of from the data itself, making it constrained by these normal cells [58]. Also, because Ginkgo is a variable bin-based approach, it is restricted to certain versions of the reference genome. For the human genome, the latest version is GRCh37.

#### SCNV

SCNV [57] automatically identifies normal cells and pools them to serve as the normal sample pair. Such identification is made by segmenting the genome by CBS with fixed-size bins, followed by computing the distance between the median read count of each segment and the average read count of the whole genome. This step also serves to remove outlier cells. SCNV then segments the genome by a generalized likelihood ratio test by pairing up each tumor cell with pooled normal cells. The parameter of segmentation is calibrated by running SCNV on (pooled) normal cells to reduce false-positive calls. This is based on the assumption that all cells are sequenced by the same platform and have similar error profiles. Note that this data-driven automated process of parameter calibration eliminates the need for modeling the noise with a certain distribution, making it suitable for all scDNAseq technologies. The other notable advantage is that although normal cells are selected by segmenting the genome into fixed-size bins, SCNV’s segmentation step does not involve bins at all. Also, since the segmentation step is bin-free, the boundaries of CNAs are at a higher resolution and the method is generalizable to data at different sequencing depths. One disadvantage of SCNV is that it requires at least 20–30 normal cells in the pool of sampled single cells [57], constraining the upstream experimental design.

### Multiple sample/cell-based methods

#### CopyNumber

CopyNumber [69] segments a single sample or multiple samples simultaneously. The segmentation strategy is objective function based, and the dynamic programming algorithm implementation makes it computationally light. It takes ∼ 2.78 h and < 1 Gb memory to process 1000 cells at 0.04 × coverage on a Intel(R) CPU E5-2650 v2, 2.60 GHz [54]. However, CopyNumber is limited in deciphering ITH because it does not pursue the absolute copy number inference after segmentation [54]. Neither does it identify which cell contains a breakpoint it infers. CopyNumber was not originally designed specifically for scDNAseq data. Therefore, although it has some pre-processing steps such as removing outlier bins by winsorizing extremely high/low signals, it does not filter out outlier cells or take into account the coverage fluctuation that is a characteristic of scDNAseq data. Finally, CopyNumber is not sensitive to CNAs occurring to small clones (clones that consist of a small number of cells). In the simulation study of [54], the recall and precision of CopyNumber were found to be generally low (< 0.4).

#### SCOPE

SCOPE [62] jointly segments all cells by a generalized likelihood ratio test proposed originally in [70] and later adopted by SCNV [57]. It controls the number of segments by a modified Bayes Information Criterion, or BIC [71]. Unique to SCOPE, data normalization considers cell-specific GC content bias, library size, and read depth, as well as the potential absolute copy number for bins of interest. This normalization requires having normal cells present as the negative control. SCOPE automatically identifies normal cells by calculating the Gini coefficient, which it uses to automatically exclude the outlier cells.

#### CHISEL

CHISEL [56] is the first allele-specific and haplotype-specific CNA detection tool for scDNAseq. It utilizes a reference-based algorithm, Eagle2, to phase blocks in each bin, with the help of either a matched normal sample or a pseudo-normal sample derived from diploid cells. It then uses an Expectation-Maximization algorithm to phase the blocks in each bin, assuming that the haplotype with a lower read count belongs to the same allele. This step produces a B-allele frequency (BAF) for each bin, which, together with the read depth, is used to cluster bins and cells. The clusters of cells help to identify the ploidy for each cell, which further enables identifying the copy number profile for each haplotype. CHISEL then groups cells into clones by hierarchical clustering. The application of CHISEL to 10 single-cell data sets in 2 breast cancer patients [56] demonstrated that, thanks in part to considering the BAF signal, it can (1) detect CNAs on some well-known breast cancer genes and (2) reconstruct a more refined tumor evolutionary scenario that has been orthogonally validated by SNVs. CHISEL is innovative in incorporating allelic signals to infer the clusters for bins and cells in addition to read depth. However, as we discussed, scDNAseq suffers from high ADO, and when alleles are separately taken into account, modeling ADO is essential for achieving high accuracy in segmenting the genome and cell clustering. From an evolutionary perspective, the result of hierarchical clustering, in this case, is not necessarily the topology of a phylogenetic tree [72].

#### SCICoNE

SCICoNE [15] is the first tool that can simultaneously infer CNA profiles for the individual cells, as well as an evolutionary tree based on those profiles. Like SCITE [28], SCICoNE produces a CNA mutation tree. SCICoNE allows for violations of an infinite-alleles assumption (see below). SCICoNE is designed specifically for low-coverage scDNAseq data and is also well applicable to human cancer data as it makes no assumption of the ploidy of the cells. In fact, it has the option to root the CNA tree at a cell whose ploidy is different from two, enabling discovering genome doubling. One limitation of SCICoNE is that it infers the breakpoints before inferring the CNAs and the underlying tree. This separation of the two processes may propagate errors made in breakpoint identification to the phylogenetic analysis.

### General considerations when using scDNAseq CNA detection methods

In this section, we discuss the eight aforementioned methods in the context of CNA sizes, CNA rates, doublets, and different experimental sequencing procedures.

CNAs that can be detected from scDNAseq data typically range in size from megabytes to whole chromosomes. All existing scDNAseq CNA detection methods, except for SCNV [57], are bin-based and assume independence among the bins. For these methods, CNA detection is done at the resolution of individual bins, regardless of the size of the actual CNA. SCNV [57], however, is an interval-based approach. It pairs up the positions of each read and tests if the interval has a significant change of the portion on the tumor cell as compared to normal cells. Such design favors large CNAs, as they have a better chance to be identified than smaller CNAs. Also, such an interval-based approach can overcome noise from single bins, and if the inference is correct, it obviates the need for chaining bins together to elucidate the actual CNAs.

CNA rates could vary significantly over time during the evolutionary history of a tumor. For example, Gao et al. [27] postulated that most CNAs are acquired early on in the evolutionary history, and after that, stable clonal expansions ensue. Not only would this variation impact phylogenetic inference (discussed below), but it could also confound CNA detection itself. Higher CNA rates would give rise to more overlapping CNAs (more than one CNA affecting the same genomic locus). These overlapping events would make it especially hard to detect the actual CNAs, even if the copy number at each bin is called correctly (which is not necessarily easy to begin with). Tools that analyze multiple samples/cells simultaneously could potentially account for part of the effects of this rate variation. For example, they could assume some large CNAs that are common to all samples (those would be the ones that occurred at the early stages of tumor evolution). None of the eight methods reviewed considers the variability of the CNA rate and models it computationally.

To the best of our knowledge, there is no CNA detection tool that can deal with doublets in an automated fashion. In [56], the authors reported a clear evidence of doublets in one of their experiments, though did not model doublets in CHISEL. As mentioned earlier, the presence of doublets might not confound the segmentation step as much as the inference of the absolute copy numbers. Thus, extra caution must be taken when interpreting CNA detection results when doublets are suspected to be in the data.

As shown in [54], scDNAseq library preparation could have a big impact on the performance of CNA detection methods. In simulating scDNAseq data that mimics the library preparation by MALBAC, DOP-PCR, and TnBC, it was shown that Ginkgo’s recall varied between 0.6 and 0.7 on these three preparation protocols, where HMMcopy had a much higher precision than Ginkgo. CopyNumber had low recall and precision across all three protocols. In summary, we recommend HMMcopy and Ginkgo for inferring CNAs from scDNAseq data. When the ploidy of the cells is not known a priori, Ginkgo was shown to be the most robust and accurate in terms of inferring the absolute copy numbers [54].

## Evolutionary modeling and analysis of CNAs

Models of evolution at the molecular level have been developed and studied by population geneticists for many decades now. In a seminal paper [73], Kimura studied two evolutionary models both of which turned out to be of great relevance to the analysis of cancer genomes: the “infinite-sites model” [74] and the “infinite-alleles model” [75]. In the infinite-sites model, it is assumed that the number of sites (nucleotides) in the genome is very large (the “infinite” in the model name) that, when coupled with a low mutation rate, whenever a new mutation arises, it is a mutation at a site that did not mutate before. This model gives rise to bi-allelic sites, where the ancestral state is often represented by 0, and the derived (mutated) state is represented by 1. The infinite-alleles model assumes that the number of possible states of a given site is very large (the “infinite” in the model name) that whenever a mutation arises at a site, it results in a new, not preexisting, allele at that site.

The infinite-sites model has already found applications in inferring evolutionary histories of cancer genomes from scDNAseq SNV data. For example, both SCITE [28] and OncoNEM [29] assume this model. Kuipers et al. [76] found strong statistical support for finite-sites model, which models the situation when a site mutates more than once in 11 out of 12 data sets. Deviations of this model have also been employed in other methods. SPhyR [31], SASC [77], and the method of McPherson et al. [78] use a Dollo parsimony model: a site is allowed to gain a mutation (go from state 0 to state 1) at most once, but is allowed to lose mutations (go from state 1 to state 0) multiple times. Departing from this assumption, methods like Sifit [30], PhiSCS [32], and SiCloneFit [33] use finite-sites models, where gain and loss of mutations are allowed to occur at any site any number of times. While all these methods use SNV data alone, the recently devised method SCARLET [79] also takes into account copy number loss as a cause of a variant loss.

When modeling CNAs, an allele at a given locus corresponds to a particular copy at that locus. Furthermore, a locus can be treated as a character whose states correspond to the possible number of copies at that locus. In this case, the evolution of such a character can rarely be modeled using the infinite-sites model, as the number of states can often exceed two. The infinite-alleles model is appropriate in the case of modeling CNAs, as it allows for capturing any number of possible alleles, but it could also be violated. Violations could occur due to back-mutations (e.g., the state of a locus mutating from state *i* to state *j* and later back to state *i*) or parallel/convergent mutations (e.g., a locus mutating to the same state more than once during its evolutionary history). While violations of the infinite-sites assumption when studying SNVs could be minimal, violations of the infinite-alleles model when studying CNAs could be ubiquitous.

A salient feature of the aforementioned methods for evolutionary analysis from scDNAseq SNV data is that sites within the same genome are assumed to be independently and identically distributed (i.i.d.). This assumption allows for defining one model of evolution that is applicable to all sites, and allows for more efficient computational inferences (i.e., taking the product over all sites when calculating the likelihood). However, while in the case of SNVs the notion of a locus is well defined (it is a site in the genome), this notion is problematic in the case of CNAs. At the highest level of resolution, copy number profiles at the level of individual genomic sites can be pursued. However, due to low coverage in scDNAseq data, analyses at this resolution would be replete with erroneous copy number estimates. At the other end of the spectrum, the entire genome is treated as a single locus. While this level of resolution provides more power for inferring the actual CNAs, especially large-scale ones that span long genomic regions (e.g., whole-genome duplication), inferences at this level quickly become intractable. Mathematical models and algorithms for genome rearrangement problems in the presence of duplication have been introduced, e.g., [80–83], some of which are being studied in the context of cancer genomics. However, these models are mostly “gene centric,” that is, they assume loci in the genome have been delineated and number of copies, as well as gene order within each genome, are unknown.

### Existing approaches relevant to the evolutionary analysis of CNAs

Dorri et al. [84] developed a method for inferring phylogenetic trees from scDNAseq copy number profiles by assuming the perfect phylogeny not on the copy numbers but rather on the breakpoints. Furthermore, SCICoNE [15] estimates a tree in addition to the inferred CNAs. The development of these methods notwithstanding, general approaches from the fields of phylogenetics, molecular evolution, and population genetics—some readily available—can be useful in evolutionary analyses of CNAs or, more generally, copy number profiles. In this section, we review such approaches.

To the best of our understanding, partitioning a genome into bins as described above and conducting bin-based analyses are most common in CNA detection. In this case, the genome of cell *i* is represented as a sequence *S*_*i*_ of *m* numbers, where *m* is the number of bins into which the genome is partitioned, and *S*_*i*,*j*_∈{0,1,2,…} is the copy number of bin *j* in the genome of cell *i*. A big question that arises here is: What should the size of the bin be? As we discussed above, some methods use fixed-size bins, whereas others use variable-size bins, but both have their shortcomings. In the context of the gene-centric genome rearrangement methods, bins become the “genes” of interest. The next natural question is whether the bins along the genome are independent or linked.

Assuming the bins are independent makes the analyses more tractable and facilitates the use of existing tools. For example, the recent study of Gao et al. [42] made this assumption. While it has not been applied to scDNAseq CNA data, the model employed by MEDICC [85] does not assume the bins are independent of each other. While this model is more realistic than the independent-bins model, analyses under this model are computationally very challenging and heuristics developed thus far are limited in applicability to a very small number of samples [86].

The choice of a model of evolution is an essential first step to all downstream evolutionary analyses, which include the task of inferring the evolutionary history of the genomes as well as the task of inferring ancestral states so that the actual mutations are elucidated.

For the phylogenetic approach, there are two main categories of methods for phylogenetic tree inference: distance-based methods and sequence-based methods. Distance-based methods compute pairwise distances between the genomes and then build a tree from the distance matrix. The most commonly used distance-based method is neighbor-joining [87]. Sequence-based methods use the genomic sequences directly to infer the tree, assuming a model of evolution. Parsimony-based inference where the number of mutations is needed to explain the data [88–90] and likelihood-based inference where the likelihood of the tree is maximized [91] are the two most commonly used approaches in this category. To apply distance-based methods in this context, the pairwise distances among copy number profiles of individual cells can be computed using one of a variety of distance measures, and the pairwise distance matrix is then used as input to a method like neighbor-joining. An important caveat here is that distances that are based directly on the observed differences between two copy number profiles can grossly underestimate the true pairwise distances (the true distance between two cells should reflect the sum of the number of CNAs on the paths from the cells’ common ancestor to those two cells). To address this problem, a *distance correction* can be applied; however, distance correction requires knowledge of a stochastic model of evolution of copy numbers. An additional benefit of deriving such stochastic models is that they enable maximum likelihood and Bayesian phylogenetic tree inferences. Maximum parsimony inference assuming independent bins can be done in a straightforward manner using the algorithms of [88–90]. By “straightforward,” we mean that existing methods apply directly. For example, in [42], Gao et al. built a maximum parsimony tree using a parsimony ratchet algorithm implemented in the R package phangorn [92]. For computing the likelihood of a tree, on the other hand, one way to do this is to define a Markov model of copy number evolution and then apply Felsenstein’s algorithm [91] to compute the likelihood of a given tree.

From the fields of molecular evolution and population genetics, models of tandemly repeated DNA, especially microsatellites, and multigene families, are very relevant to evolutionary modeling of CNAs. Litt and Luty [93] introduced *microsatellites* to describe arrays of short (eight nucleotides or shorter) tandemly repeated DNA. Microsatellites have been used as an evolutionary marker, where the state of a microsatellite captures the number of times the short DNA segment is repeated. One evolutionary model that is applicable to microsatellites is the aforementioned infinite-alleles model. This model allows for mutation in the microsatellite marker between any two possible states. An alternative evolutionary model is the *stepwise mutation model* (SMM). In this model, discrete states are ordered and a mutation can change the copy number to one of those states. Treating the different copy numbers at a locus similarly to the states of a microsatellite, the infinite-alleles model and SMM, along with methods for inference under them can be applicable to some evolutionary analyses of copy number profiles.

**Fig. 3:**
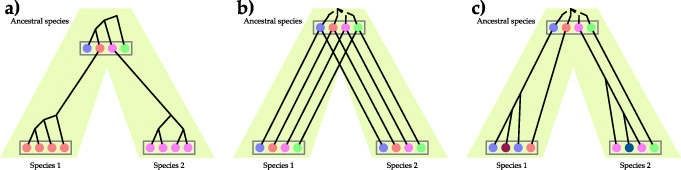
Three modes of evolution of multigene families. **a** Concerted evolution. **b** Divergent evolution. **c** Evolution by birth and death process. (Reproduced from [95])

Finally, following Ohno’s seminar work on gene duplication [94], much work has been done on understanding and modeling gene duplication in the evolutionary community. A *multigene family* is a set of genes (from the genomes under study) that have all evolved from a common ancestral gene by speciation events (resulting in orthologous genes) and duplication events (resulting in paralogous genes). Ota and Nei [95] described three different modes of evolution of gene families: *concerted evolution*, *divergent evolution*, and evolution by *birth and death* process; Fig. 3. In concerted evolution, the members of the multigene family evolve in a concerted manner, rather than independently, and gene conversion and unequal crossing-over play important roles in this model. As CNAs are often discussed in the context of somatic cells, the concerted evolution model is not of much relevance. In divergent evolution, different members of the multigene family are related by descent from the ancestral copies, but have diverged gradually, thus developing new functions. The birth-death model of multigene families is probably the most relevant of the three to evolutionary analysis of copy number profiles. In this model, a gene duplicates (birth) or is lost (death) resulting in the expansion and shrinking of the size of the family in different species. Computational methods for inferring evolutionary histories of genes under this model or for inferring evolutionary histories of species (in a data set where samples from multiple organs in the same patient are obtained, the population of cells from a single organ can be designated as a “species”) given a set of multigene families (e.g., [96]) could potentially be applicable to CNA evolutionary modeling and analysis. Here, the sequence data of the multigene families can be used directly.

To the best of our knowledge, the only methods that exist for inferring evolutionary histories from scDNAseq data while not assuming independent bins are CHISEL [56] and SCICoNE [15]. In particular, CHISEL [56] reconstructs its phylogenetic tree considering CNAs on contiguous bins and overlapping CNAs on haplotype-specific CNAs. Like MEDICC [85], CHISEL assumes the minimum number of events and reconstructs the most parsimonious tree under a model of interval events.

Finally, once an evolutionary tree is inferred, ancestral copy number profiles can be estimated for the internal nodes of the tree from the profiles of the sequenced cells at the tree’s leaves. This task is computationally much easier under the independent-bins model. Ancestral state reconstruction in a maximum parsimony manner can be accomplished using the algorithms of [89, 90] (e.g., see [97]) whereas likelihood-based inference of ancestral states can be accomplished using a simple modification to Felsenstein’s algorithm [91], as discussed in [98]. As in the case of tree inference, maximum likelihood ancestral state reconstruction requires an explicit stochastic model of copy number evolution. Ancestral state reconstruction under models that deviate from the independent-bins assumption is an open research area.

## Simultaneous inference of CNAs and evolutionary trees

The current practice in evolutionary analyses aimed at identifying CNAs and elucidating ITH is to first estimate copy number profiles for the cells under study and then infer the evolutionary tree along with CNAs and hypotheses about ITH. While aiding with computational feasibility of the analyses, this two-step analytical pipeline has its shortcomings. First, copy number profiles estimated from the data have errors in them (in the form of incorrect copy numbers). When left unaccounted for, these erroneous copy number profiles could have an impact on the accuracy of the inferred tree and would definitely impact the accuracy of ancestral state reconstruction and postulated CNAs. Furthermore, the cell-lineage tree imposes important constraints on the evolution of the copy number profiles that originate from the temporal dependencies of the cells as they descend from their most recent common ancestral cell. Cells that are closely related on the tree are expected to have more similar copy number profiles than cells that are more distantly related. Furthermore, the tree shape coupled with its branch lengths allows for quantifying the probability of observing certain profiles. For example, longer branches are expected to result in more accumulated changes than shorter branches. All these aspects combined imply that the evolutionary tree can provide a powerful framework within which to call copy number profiles, including for the sequenced cells under study. The tree acts as a “regularizer” that controls complexity as measured by the number of CNAs inferred. This observation yields a method for assessing CNAs detected from biological data sets where the ground truth is unknown. For example, given the copy number profiles at the leaves of a tree, ancestral state reconstruction is conducted, and CNAs along each branch in the tree are computed for each bin. Then, the number of copy number changes across the entire tree is computed for each bin, a distribution is plotted, and bins that deviate significantly from the mean are inspected for potential error in the copy number profiles in the input. We illustrated this approach in [54].

Perhaps most interestingly in this area is the approach of simultaneously inferring the evolutionary tree and copy number profile from the raw read-count data, which is the approach taken by SCICoNE [15]. This approach obviates the need to account for error in estimated copy number profiles and at the same time uses the tree to aid in the copy number inference task itself. Furthermore, SCICoNE does not make the independent-bins assumption. Instead, it infers the events that were formed of continuous bins in the underlying model. Incorporating more realistic models of genome evolution like SCICoNE [15] and CHISEL [56] under CNAs is one direction for future research.

## Conclusions

In this paper, we reviewed eight methods that have been developed or applied to CNA detection from scDNAseq data. In our review, we outlined the seven general steps that a CNA detection pipeline follows, and discussed the eight methods in light of these steps. For each method, we highlighted its advantages and limitations, paying special attention to the applicability of the method to scDNAseq data from cancer genomes. Two important outcomes of CNA detection are segmentation of the genome into non-overlapping regions with different copy numbers and inference of the actual copy numbers of the different segments. We reviewed the three different approaches to segmentation and discussed for each of the eight methods which of these two outcomes the method is capable of producing.

We also reviewed the tasks of evolutionary tree inference and ancestral state reconstruction. While the infinite-alleles model is more appropriate for data with CNAs than its counterpart for SNV data (the infinite-sites model), violations of this model could be rampant. Models of evolution of copy number profiles are in their infancy. Currently, the genome is partitioned into bins, and those bins are either assumed to be independent or have some limited dependencies. For the independent-bins model, standard phylogenetic inference methods are applicable almost directly, where only the models of copy number evolution within a bin potentially differ from the standard models of evolution in phylogenetics. When bins are assumed to have dependencies among them, the computational tasks become much more involved, and hardly any methods currently exist.

Last but not least, we identified simultaneous inference of the evolutionary tree and copy number profiles from the raw read-count data as a promising direction for future research. This approach would be computationally more challenging, even under an independent-bins model, but the gains in accuracy will be significant enough to justify pursuing it. Given the large amounts of scDNAseq data that are becoming available for CNA detection, developing novel techniques to achieve scalability of evolutionary analyses is imperative.

Finally, with the promises of scDNAseq come challenges from uneven and low sequencing coverage that cause erroneous CNA calls. One direction to enhance CNA detection accuracy is to create “pseudo-bulk” data by in silico pooling multiple cells together and then use the “pseudo-bulk” data to improve CNA resolution and accuracy. Another direction, which is more on the sequencing side, is to perform bulk sequencing in parallel to scDNAseq from the same tumor sample. However, analyses of such data must account for potential sampling bias, where the cells in the scDNAseq data are not necessarily representative of those in the bulk sequencing data, and vice versa. Another issue that must be accounted for is that bulk sequencing is insensitive to very rare clones and thus may misguide scDNAseq-based analyses to drop the signals from the cells that belong to such clones [18, 43, 50].

## Supplementary information

**Additional file 1** Review History.
