# Supplementary material for: Methods for copy number aberration detection from single-cell DNA-sequencing data
Source: Genome Biol. 2020 Aug 17;21:208. doi: 10.1186/s13059-020-02119-8 (PMC7433197; doi:10.1186/s13059-020-02119-8)
Supplement: Supplementary file 1 — Additional file 1 Review History. [file 13059_2020_2119_MOESM1_ESM.docx]

Review History

**First round of review**

**Reviewer 1**

**Comments to author:**

The authors have collated and described tools for learning copy number aberrations from single-cell sequencing, an important and timely topic for cancer research. The description of the general steps is well presented.

The authors have then carefully collected the methods into different groups and highlighted their different aspects. Given the variety of approaches, this is a formidable task which the authors have handled

well, though the text describing the methods could benefit from a bit of reworking to improve the flow. One question is whether it might be better to extract the segmentation part of the different algorithms as a separate section, rather than a description for each algorithm. Of course, some algorithms have this as in integral part of the copy number calling.

The sections on tree inference, on the other hand are less of a review and more a collection of the authors' thoughts, and seemed a little out of place. This could be heavily condensed.

One question is the relationship between this paper and the previous preprint of the authors benchmarking different tools. The benchmark and the review are two sides of the same coin, and including benchmarking of the reviewed methods would add a lot of value to the current manuscript.

More minor points:

In the first line of the introduction, ITH is described as a confounding factor and a clarification of how ITH is confounding and the effects it has would be welcome.

The second paragraph of the intro discusses generally the topics exemplified by scDNA SNV methods, so it would make sense to move that overview earlier. In particular the statement about performing mutation calling and tree inference jointly to improve performance is supported by works like SCIPhi which do so.

In the segmentation section of the overview of the steps, some references would be good for each approach. The objective function has much more detail than the other approaches, but still not enough to be comprehensive, so it might be preferable to summarise it to the level of the other two approaches.

In the introduction to the methods, for example SCNV, seqCBS is mentioned for several methods, but not introduced itself. Also for SCNV, how would a damped sine wave help identify the ploidy?

AneuFinder and HMMCopy are fairly similar, and their sections could be together.

For chisel, BAF is not defined. And EM is not used outside the definition. Sometimes it is capital, sometimes lower case.

The testing and results of Genome Research 27 1885-1894 (2017) could add to the discussion of the infinite sites for SNVs and how minimal their effect is.

For sites being iid, the authors should distinguish between the data noise being iid (given the copy number or mutation state) and the sites being independent (that they can mutate regardless of each other). It is the dependence between sites due to copy number segments changing that is the issue for sequence phylogeny models (like the Felsenstein) algorithm so that the likelihood cannot be factored over the sites. Independence across cells is still a reasonable assumption.

The formula for matrix exponential does not serve any purpose.

Figure 1e is not very instructive.

In Figure 2a, isn't the sliding window predefined and not based on the segmentation points? Also the test would be across the segments?

**Reviewer 2**

**Comments to author:**

In this review paper, Fan et al. survey copy number callers for single-cell DNA sequencing data. The authors identify seven steps that are common to such approaches, and classify them according to differences in the steps as well as other features. Overall the paper is well written and the topic is timely. This review lays out some good directions for future work and will be interest to the community, both to method developers and users. I have one major comment and several minor points.

1. Single-cell sequencing technologies

While this review does a good job surveying existing approaches, few details are given about the input these methods take. It would be good to discuss current single-cell sequencing technologies (scDNAseq based on DOP-PCR, 10X Genomics Chromium, Mission Bio, pseudobulk sequencing, etc.). In particular it would be good to list their differences, error profiles, limitations and subsequent implications for copy number callers. This would provide the proper context for the rest of the review paper.

Minor comments:

* Paper mentions that there current single-cell phylogeny inference methods for SNVs do not use CNA data. There is a recent method that does this: SCARLET (RECOMB 2020/Cell Systems).

* A table similar to Table 1 for CNP distance measures would be very helpful

* Define B-allele frequency

* Define Gini coefficient

* CNA or CNV -- good to indicate what the difference is between these two.

* It would be good to define what a false negative/positive is (mismatch in breakpoints, copy number?)

* Chisel or CHISEL?

**Reviewer 3**

**Comments to author:**

The authors of this manuscript have made seminal contributions, methodological and clinical, in the field of single-cell cancer genomics, and they really have to be praised for that. Here, they present a review of single-cell copy number calling, a timely contribution to the field. I found the review interesting, but at the same time I felt that several aspects could be improved.

** Major comments **

1) Scope

Somehow, my impression that the review addresses many things at variable depths. The title refers to detection of copy number alterations (CNAs) in single-cell DNA sequencing data, but the text seems to go in multiple directions.

For example, the review mixes bulk and single-cell approaches, and it is not always clear to which scenario a given passage is referring to. I believe that bulk CNA detection has been addressed elsewhere in multiple occasions, and that focusing on single-cell CNA (scCNA) detection, as the title suggests, would increase readability. There are plenty of issues to discuss just on this.

I believe the text refers exclusively to human cancer cells. Several issues would be different in somatic and/or non-human tissues (ploidy, purity, CN rate, genomic reference, genome structure and size, etc.). I would clarify at the start that the focus is human cancer, also in the title.

I suggest each of the methodological steps of scCNA calling is presented along with a discussion of how the single-cell genomics particularities affect (or not) each of them. Also, a figure including an example of inferred scCNA profiles for a group of cells could be useful.

The manuscript not only addresses CNA detection and evolution but also CNA phylogenetic analysis. While a discussion of how CNA evolve is necessary to develop realistic detection algorithms, phylogenetic analyses is a related but different topic that might be better addressed in a separate review. If the authors believe that it should still be included, then I believe the contents should be modified (see below).

2) Style

In general, my impression is that the depth of the argumentation varies perhaps too much. The text combines more or less shallow paragraphs with some very technical descriptions, already available in the programs' documentation, which in my opinion are not important in a journal like this.

Also, I felt that there was a bit of redundancy when comparing the introduction (saying what is going to be done), results (showing what was done) and conclusions (which says what was done). Perhaps the text could be streamlined a bit.

In conclusions, it would be ideal to provide the user with some recommendations regarding experimental design, data analysis and interpretation caveats, for the different scenarios in which scCNA calling might be of interest. This final section should highlight the main insights, offering a guideline for the user to choose the best method for the question at hand.

3) Single-cell genomics

I believe that in a review of scCNA a section on the different biases that affect single-cell sequencing data is mandatory. The particularities of single-cell genomics are extremely relevant for CNA detection, as very noisy profiles can result from the amplification step (to a different step depending on the particular amplification methodology). In this type of data, allelic imbalance, allelic dropout, doublets and others are factors that need to be considered for scCNA detection. Variable sequencing depth and amplification recurrence are key, copy numbers are integers, there can be contamination, there might be cell population structure. One could use consensus strategies, or combine the single-cell data with the bulk. In my opinion, all this needs to be discussed in depth, comparing also the advantages/disadvantages of the different experimental procedures to obtain good CN calls, together with a discussion, later on, on how each method deals with these issues.

5) Describing and comparing methods

Ideally, one would compare tools identifying potential scenarios in which some would be more appropriate than others (depending on the data type, the size of the data, the CN rate, the question at hand, etc.). While Table 1 is nice and informative, I found the description of the methods often too technical, feeling sometimes as an enumeration of features extracted from the program documentation. In my opinion, the description of the methods should focus on helping the user to make choices. For example, I believe that a typical reader of this journal read user will not really care whether some program uses a Cauchy or a Dirichlet distribution, or a Baum-Welch algorithm, per se. Instead, I expect readers to care more about the biological assumptions behind the use of these distributions or algorithms. Some of these aspects are already discussed, like independence of the events, ploidy, marginal/joint calling, but it would be also interesting to know how methods deal with different CN sizes, CN rates, different experimental procedures for the amplification (if there is such a thing, as direct library preparation methods exist; they should be discussed), which type of genomic reference they can use, whether they can use target/WES/WGS data, or the purity requirements of the samples. It would be nice also to mention whether methods offer some type of statistical uncertainty measurement.

Failures of the methods are mentioned, but often without demonstrating why this the case, and without citations. If a method has limitations, maybe one could explain why and how they affect user's choices.

Computational demands and scalability are also key, and the user should learn which dataset sizes a method can deal with, in a grossly manner if you want. I assume some methods are lighter and faster than others. In this regard, and in general, it would be very informative for the reader to see the results of the analysis of at least one real data set with the different callers and compare the CN calls, including runtime.

Also, it would be nice to summarize here the main conclusions arising from empirical and in silico benchmarks of scCNA calling. For example, Ginkgo was compared with bulk CN calling algorithms, and there might be now other comparisons out there. Also, the authors have a preprint on in silico benchmark of scCNA calling, and perhaps could summarize here the main conclusions.

Finally, new methods have appeared very recently for simultaneous inference of CNA call and phylogenetic tree, that probably should be now included in this review: https://www.biorxiv.org/content/10.1101/2020.04.28.065755v1.

4) CNA evolution

A section on how CNAs take place is important to understand which detection methods are more realistic, and therefore putatively superior. Such a section could be located at the very beginning.

A section explaining how CNAs evolve is indeed necessary, and the authors have made a very good job discussing key issues like recurrence and non-independence of CNA events at the beginning of the section "Evolutionary Modeling and Analysis of CNAs". However, as I already mentioned, I believe the phylogenetic analysis of CNAs is a related but different topic, even if one could use a tree to help CNA detection, this is something that can be just mentioned in a review like this in principle focused on detection (according to the title). However, if you insist on including a whole section on CNA phylogenetic analysis, then, in my opinion, it should be changed quite a bit.

For example, I do not think that one needs to refer to general phylogenetic concepts available elsewhere (there are multiple tumor phylogenetic reviews already), like parsimony or distance-based method, or to explain here P=e^Qt. Instead, I would refer to what is known already about copy-number-like scenarios in organismal molecular evolution, and that could be used for scCNA. For example, one could refer to existing phylogenetic models for gene family evolution, using gene counts or sequences, and for microsatellites (stepwise mutation models are very relevant here anyway), and in general, to birth-death models in molecular evolution. There is ample literature on this, some goes back to the 70's and to Kimura, and in my opinion it would much informative to discuss how they could be leveraged to develop better CNA calling and phylogenetic methods, than to show simple distance calculations that have been superseded long time ago.

** Minor specific comments **

[page, line]

2, 31: CNV is not defined. Please clarify the difference, if any, between CNA and CNV. These terms are used in different, often contradictory, ways in the literature.

2, 32: application to scDNAseq data of *human* cancer genomes.

4, 43: remove "it has been observed that"

4, 44: "GC correction starts with modeling the change of (log normalized) read count with respect to GC content, either parametrically by a polynomial function, or non-parametrically by LOESS regression [15,22,27]." => I believe this is unnecessary detail, and instead I would write something like "The GC correction starts with modeling the change of read count with respect to GC content" . I would revise the whole text accordingly as there are multiple statements which seem too technical. I would stress instead the assumptions and capabilities behind, and what the user should or could do about it.

4, 48: "The data to be analyzed is used to study the model," => rewrite, maybe "The GC correction is specific for each cell, as…"

5, 40: "Both low coverage and large Gini coefficient indicate potential failed library preparation" => I would explain what is the Gini coefficient, what is used for (depth heterogeneity), and hot it relates to CNA calling.

6, 18: "The first term is the distance between the underlying piecewise constant function and the given data. The second term is the number of the changes, which is usually an L1 norm total variation term. The two terms are often combined by a Lagrange multiplier. Numerical methods are often applied to find the piecewise constant function that minimizes the objective function." => to me, this is another example of the type of paragraphs that need to be removed or rewritten; they are too technical and I believe add not relevant information for the reader.

6, 31: "Twenty-nine methods … are reviewed in this section", and then ten lines below "In the following section, we review the seven methods". (but as I mentioned, I would remove from this review methods that no specific for single cells)

6,62: "It models the emission probability of each state by Gaussian distribution, whose mean and the inverse of variance are modeled by Gaussian and Gamma prior distributions, respectively. It models the transition probabilities by a Dirichlet distribution. The probability of a bin being an outlier is modeled by Beta distribution. HMMcopy estimates the state sequence and parameters simultaneously by blocked Gibbs sampling" => Again, I believe this type of information is not useful for the readers of Genome Biology. Instead I suggest focusing on biological assumptions, robustness to single-cell genomics biases, and in general to advantages/disadvantages of the method for scCNA. Can HMM be really applied to single-cells? How does it deal with, for example, with biases like ADO? There are many paragraphs like this before and after in the text, I suggest a thorough rewriting of them.

6, 32: remove "etc" and enumerate the specifics.

6,55: 16,698 single cells. => which type of cells? human?

7, 23: 54 *human* normal diploid cells?

7, 25: What is a "CBS-based approach"?

7, 31: "Ginkgo also clusters the cells, infers the phylogenetic tree *of* the cells ."

7, 34: "The limitations of Ginkgo include the fact that the list of bad bins is learned from a fixed set of normal cells, and its GC correction does not incorporate each bin's copy number. => In this type of statements (there are a few like this) I suggest you try to explain the reasoning and consequences of these observations. Why are these limitations bad?

7, 61: What is the L2 norm? do we really need this info?

9,18: "important to be inferred simultaneously. In addition, maximum parsimony criterion can be used to more accurately infer the tree than hierarchical clustering." => can you justify this statement?

13, 30: Is not reference 64 this same manuscript? (paper self-citation?)

I think a review like this could be useful, but I suggest a few changes of style and focus. You are welcome to contact me for any clarification.

David Posada (dposada@uvigo.es)

**Authors Response**

**Point-by-point responses to the reviewers’ comments:**

Reviewer #1 Comments:

The authors have collated and described tools for learning copy number aberrations from single-cell sequencing, an important and timely topic for cancer research. The description of the general steps is well presented.

The authors have then carefully collected the methods into different groups and highlighted their different aspects. Given the variety of approaches, this is a formidable task which the authors have handled well, though the text describing the methods could benefit from a bit of reworking to improve the flow. One question is whether it might be better to extract the segmentation part of the different algorithms as a separate section, rather than a description for each algorithm. Of course, some algorithms have this as an integral part of the copy number Calling.

*We have now reorganized the writing. The first five steps are really "data wrangling" steps that involve cleaning the data, and are not necessarily specific to CNA detection in general, as much as they're relevant to scDNAseq data. The last two steps (segmentation and calling absolute copy numbers) are really the core of CNA detection. So, we now have two subsections, one dedicated to the first five steps, and one to the last two, with segmentation and calling absolute copy numbers described separately and in more detail.*

The sections on tree inference, on the other hand are less of a review and more a collection of the authors' thoughts, and seemed a little out of place. This could be heavily condensed.

*Following the comments of this reviewer and Reviewer #3, we have heavily revised the section. Evolutionary analysis has become very big in cancer genomics. While evolutionary analyses of SNV data have become common, this task is not well developed for CNA data. Our goal is to draw upon the existing literature to highlight directions that could be fruitful to pursue in this field. We hope the new revised section is to the reviewers' satisfaction (we ourselves feel it's much better now that it's been revised in response to the reviewers' feedback, and we are very thankful).*

One question is the relationship between this paper and the previous preprint of the authors benchmarking different tools. The benchmark and the review are two sides of the same coin, and including benchmarking of the reviewed methods would add a lot of value to the current manuscript.

*We have discussed this with the Editor and have taken two actions. First, we now provide reference to the benchmarking study and refer to our findings there in the relevant sections. Second, we have withdrawn the benchmarking manuscript from the journal where it was under review and will submit it to Genome Biology. While we are not opposed to combining the two into a single manuscript, I believe the reviewers and Editor will agree with us that combining them would create a massive manuscript most of which would have to be relegated to a supplementary materials section. The benchmarking study, for example, reports on a new simulator we developed specifically for CNAs in scDNAseq data. In addition to the excessive length of a single combined manuscript, we feel that a combined manuscript will have too many important messages that shouldn't all be part of a single manuscript, or else they'd be lost on the reader. However, once the benchmarking study is submitted to GB, we're happy to hear the Editor's and reviewers' thoughts on options for how to handle both manuscripts.*

More minor points:

In the first line of the introduction, ITH is described as a confounding factor and a clarification of how ITH is confounding and the effects it has would be welcome.

*We edited the text explaining why ITH is confounding and the effects it has in the first paragraph of the text.*

The second paragraph of the intro discusses generally the topics exemplified by scDNA SNV methods, so it would make sense to move that overview earlier. In particular the statement about performing mutation calling and tree inference jointly to improve performance is supported by works like SCIPhi which do so.

*We intended to link simultaneous calling of mutation and the phylogenetic tree to both SNV and CNAs in our second paragraph. We now added the reference to two methods in this paragraph, SCIPhi on SNV, and SCICoNE on CNA, both of which infer mutation and the tree jointly.*

In the segmentation section of the overview of the steps, some references would be good for each approach. The objective function has much more detail than the other approaches, but still not enough to be comprehensive, so it might be preferable to summarise it to the level of the other two approaches.

*We have made the discussion of all three approaches more uniform in coverage.*

In the introduction to the methods, for example SCNV, seqCBS is mentioned for several methods, but not introduced itself. Also for SCNV, how would a damped sine wave help identify the ploidy?

*This comment is probably no longer relevant. As Reviewer #3 suggested, our writeup was too heavy on the technical details of methods (as he wrote, some of the details belong in a user manual of the method). We have now revised the writeup heavily so as to focus on the methods from the perspective of a user, not a technical developer.*

AneuFinder and HMMCopy are fairly similar, and their sections could be together.

*We agree with the reviewer and have combined both methods in a single section.*

For chisel, BAF is not defined. And EM is not used outside the definition. Sometimes it is capital, sometimes lower case.

*We have addressed these points.*

The testing and results of Genome Research 27 1885-1894 (2017) could add to the discussion of the infinite sites for SNVs and how minimal their effect is.

*We have addressed this in the paragraph discussing the infinite-sites assumption in SNV analyses.*

For sites being iid, the authors should distinguish between the data noise being iid (given the copy number or mutation state) and the sites being independent (that they can mutate regardless of each other). It is the dependence between sites due to copy number segments changing that is the issue for sequence phylogeny models (like the Felsenstein) algorithm so that the likelihood cannot be factored over the sites. Independence across cells is still a reasonable assumption.

*We have clarified this point.*

The formula for matrix exponential does not serve any purpose.

*We have revised this section heavily and the formula is no longer there or needed.*

Figure 1e is not very instructive.

*This figure is just to illustrate how the Gini index is computed. While it's very simple, we would like to keep it so that the figure thoroughly captures all seven steps.*

In Figure 2a, isn't the sliding window predefined and not based on the segmentation points? Also the test would be across the segments?

*Yes, and we have clarified this point in the caption of Fig. 2.*

Reviewer #2:

In this review paper, Fan et al. survey copy number callers for single-cell DNA sequencing data. The authors identify seven steps that are common to such approaches, and classify them according to differences in the steps as well as other features. Overall the paper is well written and the topic is timely. This review lays out some good directions for future work and will be interest to the community, both to method developers and users. I have one major comment and several minor points.

1. Single-cell sequencing technologies

While this review does a good job surveying existing approaches, few details are given about the input these methods take. It would be good to discuss current single-cell sequencing technologies (scDNAseq based on DOP-PCR, 10X Genomics Chromium, Mission Bio, pseudobulk sequencing, etc.). In particular it would be good to list their differences, error profiles, limitations and subsequent implications for copy number callers. This would provide the proper context for the rest of the review paper.

*We thank the reviewer for this great comment. We have now added a new section titled "Particularies of scDNAseq" where we discuss eleven existing single-cell sequencing technologies including MDA, MALBAC, DOP-PCR, 10X, Mission Bio, etc. Furthermore, we have added Table 1 that lists their particularities and whether they are suitable for CNA calling or not. We also discussed the implications of doublets in the same section. As for using "pseudo-bulk" to improve CNA detection, we added it in the final paragraph of the Conclusions section, along with the caveats of joint analysis of bulk sequencing and scDNAseq data.*

Minor comments:

* Paper mentions that the current single-cell phylogeny inference methods for SNVs do not use CNA data. There is a recent method that does this: SCARLET (RECOMB 2020/Cell Systems).

*We agree with the reviewer, and have now added this point to the paragraph where we discuss SNV methods.*

* A table similar to Table 1 for CNP distance measures would be very helpful

*The entire section on evolutionary analysis has been revised.*

* Define B-allele frequency

*We added the definition.*

* Define Gini coefficient

*We moved the description of Gini coefficient next to the first appearance.*

* CNA or CNV -- good to indicate what the difference is between these two.

*We explained CNA in the abstract. Since we removed the review of the methods that have been applied to detect CNV as suggested by Reviewer #3, CNV becomes not the focus of this paper and we avoided using the term CNV in the main text. In general, CNA is a term that's applied to copy number variants in cancer genomes, whereas the community uses CNVs for copy number variants in healthy cells. We are not advocating this naming, but are merely using it according to the custom in the cancer genomics community.*

* It would be good to define what a false negative/positive is (mismatch in breakpoints, copy number?)

*We added the definition in the text. They refer to missing/false calling of breakpoints in the context.*

* Chisel or CHISEL?

*We capitalized all occurrences of CHISEL.*

Reviewer #3:

The authors of this manuscript have made seminal contributions, methodological and clinical, in the field of single-cell cancer genomics, and they really have to be praised for that. Here, they present a review of single-cell copy number calling, a timely contribution to the field. I found the review interesting, but at the same time I felt that several aspects could be improved.

** Major comments **

1) Scope

Somehow, my impression that the review addresses many things at variable depths. The title refers to detection of copy number alterations (CNAs) in single-cell DNA sequencing data, but the text seems to go in multiple directions.

For example, the review mixes bulk and single-cell approaches, and it is not always clear to which scenario a given passage is referring to. I believe that bulk CNA detection has been addressed elsewhere in multiple occasions, and that focusing on single-cell CNA (scCNA) detection, as the title suggests, would increase readability. There are plenty of issues to discuss just on this.

*We thank the reviewer for this comment and agree with it. We have now refocused the review on CNA detection from scDNAseq and removed all discussion of bulk sequencing data except when relevant to the methods here. We also revised the tables that describe the methods.*

I believe the text refers exclusively to human cancer cells. Several issues would be different in somatic and/or non-human tissues (ploidy, purity, CN rate, genomic reference, genome structure and size, etc.). I would clarify at the start that the focus is human cancer, also in the title.

*The review is definitely about cancer genomes, and the use of copy number aberrations (CNA) rather than copy number variants (CNV) is aimed at emphasizing this point. In terms of applicability of the methods beyond human cancer genomes, we don't feel comfortable ruling out other cancers, as the method developers might find our review to be biasing against their methods. For example, while ploidy could differ in non-human genomes, this could be an issue of the performance of the method rather than general applicability or lack thereof.*

I suggest each of the methodological steps of scCNA calling is presented along with a discussion of how the single-cell genomics particularities affect (or not) each of them.

*We have done this by adding a new section on scDNAseq particularities and their relevance/impact for each of the steps.*

Also, a figure including an example of inferred scCNA profiles for a group of cells could be useful.

*At the end of the describing "Calling the absolute copy numbers", we referred the readers to an example of such figure in a reference.*

The manuscript not only addresses CNA detection and evolution but also CNA phylogenetic analysis. While a discussion of how CNA evolve is necessary to develop realistic detection algorithms, phylogenetic analyses is a related but different topic that might be better addressed in a separate review. If the authors believe that it should still be included, then I believe the contents should be modified (see below).

*The Editor has requested that we keep this section and we agree. Having said this, the reviewer's comments and feedback are tremendously helpful and we have revised the section heavily and tried our best to draw upon methods from molecular evolution and population genetics that are relevant in this context. We appreciate the reviewer's feedback.*

2) Style

In general, my impression is that the depth of the argumentation varies perhaps too much. The text combines more or less shallow paragraphs with some very technical descriptions, already available in the programs' documentation, which in my opinion are not important in a journal like this.

*We agree and have revised the method description to remove technical details and focus on the user's perspective.*

Also, I felt that there was a bit of redundancy when comparing the introduction (saying what is going to be done), results (showing what was done) and conclusions (which says what was done). Perhaps the text could be streamlined a bit.

*We have addressed this, mainly by removing the last paragraphs from the Introduction. We kept the text in the Conclusions since it summarizes the main points.*

In conclusions, it would be ideal to provide the user with some recommendations regarding experimental design, data analysis and interpretation caveats, for the different scenarios in which scCNA calling might be of interest. This final section should highlight the main insights, offering a guideline for the user to choose the best method for the question at hand.

*We hope that by addressing all of the above comments, the review is now more along the lines of what the reviewer suggests.*

3) Single-cell genomics

I believe that in a review of scCNA a section on the different biases that affect single-cell sequencing data is mandatory. The particularities of single-cell genomics are extremely relevant for CNA detection, as very noisy profiles can result from the amplification step (to a different step depending on the particular amplification methodology). In this type of data, allelic imbalance, allelic dropout, doublets and others are factors that need to be considered for scCNA detection. Variable sequencing depth and amplification recurrence are key, copy numbers are integers, there can be contamination, there might be cell population structure. One could use consensus strategies, or combine the single-cell data with the bulk. In my opinion, all this needs to be discussed in depth, comparing also the advantages/disadvantages of the different experimental procedures to obtain good CN calls, together with a discussion, later on, on how each method deals with these issues.

*We have added the section "Particularies of scDNAseq" to discuss these issues. We discussed how the eight reviewed methods deal with these issues. And, while it is still in its infancy in the context of CNAs, we now discuss the notion of pseudo-bulk and how it can be used (mainly in the Conclusions section).*

5) Describing and comparing methods

Ideally, one would compare tools identifying potential scenarios in which some would be more appropriate than others (depending on the data type, the size of the data, the CN rate, the question at hand, etc.). While Table 1 is nice and informative, I found the description of the methods often too technical, feeling sometimes as an enumeration of features extracted from the program documentation.

In my opinion, the description of the methods should focus on helping the user to make choices. For example, I believe that a typical reader of this journal read user will not really care whether some program uses a Cauchy or a Dirichlet distribution, or a Baum-Welch algorithm, per se. Instead, I expect readers to care more about the biological assumptions behind the use of these distributions or algorithms. Some of these aspects are already discussed, like independence of the events, ploidy, marginal/joint calling, but it would be also interesting to know how methods deal with different CN sizes, CN rates, different experimental procedures for the amplification (if there is such a thing, as direct library preparation methods exist; they should be discussed), which type of genomic reference they can use, whether they can use target/WES/WGS data, or the purity requirements of the samples. It would be nice also to mention whether methods offer some type of statistical uncertainty measurement.

*We have removed all the unnecessary technical descriptions of the methods and focused more on describing the methods from the user's perspective.*

Failures of the methods are mentioned, but often without demonstrating why this the case, and without citations. If a method has limitations, maybe one could explain why and how they affect user's choices.

*We rewrote the review of the methods. Whenever applicable, we explained the reasons when stating methods' limitations and added the citations. We also explained why and how they affect users' choices.*

Computational demands and scalability are also key, and the user should learn which dataset sizes a method can deal with, in a grossly manner if you want. I assume some methods are lighter and faster than others. In this regard, and in general, it would be very informative for the reader to see the results of the analysis of at least one real data set with the different callers and compare the CN calls, including runtime.

*We now discuss the scalability for a few methods which we tested, including HMMcopy, Ginkgo and CopyNumber. The results were reported in detail in our benchmarking study that, as we discussed above, will now be submitted to Genome Biology and hopefully will be linked to this manuscript.*

Also, it would be nice to summarize here the main conclusions arising from empirical and in silico benchmarks of scCNA calling. For example, Ginkgo was compared with bulk CN calling algorithms, and there might be now other comparisons out there. Also, the authors have a preprint on in silico benchmark of scCNA calling, and perhaps could summarize here the main conclusions.

*We discussed this with the Editor. As suggested by the Editor, we now describe the main findings from the benchmarking manuscript and reference it. We have also withdrawn the benchmarking study from the other journal where it was under review and will submit to Genome Biology. We are fine with suggestions by the Editor and reviewers on how to present both manuscripts.*

Finally, new methods have appeared very recently for simultaneous inference of CNA call and phylogenetic tree, that probably should be now included in this review: https://www.biorxiv.org/content/10.1101/2020.04.28.065755v1.

*We thank the reviewer for bringing this to our attention. The field is moving very fast, and this method was posted online just a few days before we received the review. We have now included it.*

4) CNA evolution

A section on how CNAs take place is important to understand which detection methods are more realistic, and therefore putatively superior. Such a section could be located at the very beginning.

*We have added a brief description in the Introduction, but have also referred to how CNAs take place throughout the revised manuscript.*

A section explaining how CNAs evolve is indeed necessary, and the authors have made a very good job discussing key issues like recurrence and non-independence of CNA events at the beginning of the section "Evolutionary Modeling and Analysis of CNAs". However, as I already mentioned, I believe the phylogenetic analysis of CNAs is a related but different topic, even if one could use a tree to help CNA detection, this is something that can be just mentioned in a review like this in principle focused on detection (according to the title). However, if you insist on including a whole section on CNA phylogenetic analysis, then, in my opinion, it should be changed quite a bit.

For example, I do not think that one needs to refer to general phylogenetic concepts available elsewhere (there are multiple tumor phylogenetic reviews already), like parsimony or distance-based method, or to explain here P=e^Qt. Instead, I would refer to what is known already about copy-number-like scenarios in organismal molecular evolution, and that could be used for scCNA. For example, one could refer to existing phylogenetic models for gene family evolution, using gene counts or sequences, and for microsatellites (stepwise mutation models are very relevant here anyway), and in general, to birth-death models in molecular evolution. There is ample literature on this, some goes back to the 70's and to Kimura, and in my opinion it would much informative to discuss how they could be leveraged to develop better CNA calling and phylogenetic methods, than to show simple distance calculations that have been superseded long time ago.

*We appreciate the reviewer's comments very much. We have revised the section heavily and hope he finds it better written and that the exposition is more informative now.*

** Minor specific comments **

[page, line]

2, 31: CNV is not defined. Please clarify the difference, if any, between CNA and CNV. These terms are used in different, often contradictory, ways in the literature.

*We addressed at the very first occurrence of the acronyms in the abstract.*

2, 32: application to scDNAseq data of *human* cancer genomes.

*Done.*

4, 43: remove "it has been observed that"

*Done.*

4, 44: "GC correction starts with modeling the change of (log normalized) read count with respect to GC content, either parametrically by a polynomial function, or non-parametrically by LOESS regression [15,22,27]." => I believe this is unnecessary detail, and instead I would write something like "The GC correction starts with modeling the change of read count with respect to GC content" . I would revise the whole text accordingly as there are multiple statements which seem too technical. I would stress instead the assumptions and capabilities behind, and what the user should or could do about it.

*We have revised the text.*

4, 48: "The data to be analyzed is used to study the model," => rewrite, maybe "The GC correction is specific for each cell, asﾅ"

*Fixed.*

5, 40: "Both low coverage and large Gini coefficient indicate potential failed library preparation" => I would explain what is the Gini coefficient, what is used for (depth heterogeneity), and how it relates to CNA calling.

*We moved the description of Gini coefficient next to its first appearance. We also explained what it is used for and related it to CNA calling.*

6, 18: "The first term is the distance between the underlying piecewise constant function and the given data. The second term is the number of the changes, which is usually an L1 norm total variation term. The two terms are often combined by a Lagrange multiplier. Numerical methods are often applied to find the piecewise constant function that minimizes the objective function." => to me, this is another example of the type of paragraphs that need to be removed or rewritten; they are too technical and I believe add not relevant information for the reader.

*This has been rewritten.*

6, 31: "Twenty-nine methods ﾅ are reviewed in this section", and then ten lines below "In the following section, we review the seven methods". (but as I mentioned, I would remove from this review methods that no specific for single cells)

*This has changed significantly as we now focus on only 8 methods specific to scDNAseq.*

6,62: "It models the emission probability of each state by Gaussian distribution, whose mean and the inverse of variance are modeled by Gaussian and Gamma prior distributions, respectively. It models the transition probabilities by a Dirichlet distribution. The probability of a bin being an outlier is modeled by Beta distribution. HMMcopy estimates the state sequence and parameters simultaneously by blocked Gibbs sampling" => Again, I believe this type of information is not useful for the readers of Genome Biology. Instead I suggest focusing on biological assumptions, robustness to single-cell genomics biases, and in general to advantages/disadvantages of the method for scCNA. Can HMM be really applied to single-cells? How does it deal with, for example, with biases like ADO? There are many paragraphs like this before and after in the text, I suggest a thorough rewriting of them.

*We agree with the reviewer and have revised the way we describe methods significantly.*

6, 32: remove "etc" and enumerate the specifics.

*Done.*

6,55: 16,698 single cells. => which type of cells? Human?

*Yes, the cell type is human. We rewrote the paragraph entirely and this sentence is no longer relevant.*

7, 23: 54 *human* normal diploid cells?

*Done.*

7, 25: What is a "CBS-based approach"?

*We spelled out CBS and also cited the relevant paper.*

7, 31: "Ginkgo also clusters the cells, infers the phylogenetic tree *of* the cells ."

*Done.*

7, 34: "The limitations of Ginkgo include the fact that the list of bad bins is learned from a fixed set of normal cells, and its GC correction does not incorporate each bin's copy number. => In this type of statements (there are a few like this) I suggest you try to explain the reasoning and consequences of these observations. Why are these limitations bad?

*We added texts to explain the reasoning and consequences of these observations. Specifically, we explained why the bad bins learned from a fixed set of normal cells limits Ginkgo, as well as what is the consequence of conducting GC correction without incorporating each bin's copy number.*

7, 61: What is the L2 norm? do we really need this info?

*This has been revised.*

9,18: "important to be inferred simultaneously. In addition, maximum parsimony criterion can be used to more accurately infer the tree than hierarchical clustering." => can you justify this statement?

*We have rewritten these statements. The main point is that the tree produced by hierarchical clustering is not necessarily (the topology of) a phylogenetic tree.*

13, 30: Is not reference 64 this same manuscript? (paper self-citation?)

*Reference 64 (now reference [56]) is not the same manuscript as this one. It is the benchmarking paper that evaluates the three methods that have been widely applied to scDNA-seq data.*

I think a review like this could be useful, but I suggest a few changes of style and focus. You are welcome to contact me for any clarification.

David Posada (dposada@uvigo.es)

**Second round of review**

**Reviewer 1**

page 3 line 54: the degree of these artifacts can depend on the sequencing technology

page 15, line 51: SCICoNE does not have an independent bin model, in fact this is their rationale for using an event tree model rather than a cell-based phylogeny. Events are formed of contiguous bins in their model.

The manuscript has several copy-editing errors and would benefit from some proofreading

**Reviewer 3**

The authors have made a nice effort to revise the manuscript. Clearly, the scope of the article and the writing style have improved, but I have to say that the level of ambiguity in the text is still significant (see below minor comments).

The section of scDNAseq technologies is an improvement. Table 1 is the type of summary I appreciate in a review. Sections like "General Considerations When Using scDNAseq CNA Detection Methods" also make the review more useful for the reader.

** (relatively) Major comments **

1) Guideline

While now we can see more recommendations regarding experimental design, data analysis, and interpretation. I still miss a condensed guideline for the user to choose the best scCNA caller for the question at hand. Normally such a guideline is the most useful part of a review.

2) Extended concepts

For example, how does the reader exactly measure coverage heterogeneity? In particular, I miss a more detailed explanation of "phase", and allele-specific CNA calls, which indeed they are fundamental for improved CNA evolutionary models.

3) Relevant preprint

A very relevant preprint that should be discussed in depth is "Efficient Bayesian inference of phylogenetic trees from large scale, low-depth genome-wide single-cell data" by Dorri et al.; bioRxiv preprint doi: https://doi.org/10.1101/2020.05.06.058180.

4) Running times

Running times are now included for some, but not all, methods. I still think that it would be very informative for the reader to see the results of the analysis of at least one real data set with the different callers and compare the CN calls, including runtime.

5) Publishing strategy

About publishing the review and the benchmark as two different papers side-by-side, I am OK with it.

** Minor specific comments **

[page, line]

Abstract: "Results: In this review, we review eight methods that have been developed for detecting copy number aberration (CNA)" => add "in scDNAseq data".

2, 6: "Moreover, quite a few methods that have been applied to detect CNAs from scDNAseq data were "borrowed" from other types of data" => methods cannot be borrowed from data. One can see what you mean but this sounds too informal; this statement needs to be rewritten.

2, 9: "Since DOP-PCR [17] and TnBC [18], the single-cell DNA amplification protocols generating data suitable for CNA detection, are limited in terms of their coverage depth and uniformity, it is important to study the existing methods in terms of how well they can be adapted to scDNAseq data." => some words seem to be missing

2, 18: "Finally, while SNVs occur to only one nucleotide" => sounds strange to me

2, 19: define "focal region".

2, 19: "The former is a result of genomic instability due to segregation error during anaphase [20], whereas the latter is due to the gain of selective advantage during tumor progression [21-23]."=> I am not sure if former here refers to SNVs, or to the whole chromosomal events, I assume it is the latter. Most importantly, that CNAs "are due" to the gain a of a selective advantage does not make sense to me. Selection could never be the "cause" of a change. Moreover, a CNA can become abundant and even clonal just by chance (i.e., neutral mutations can fix in populations with more or less probability depending on multiple factors). As written, it seems that all CNAs we see in a tumor offer selective advantages, which is clearly not the case.

2, 59: "far from enough" could be understood as too little or as too much. It is easy to be precise by writing "not enough"

3, 7: remove "etc.", if you know more methods, enumerate them. Alternatively, if you just want to highlight some examples, use "e.g.".

3, 10: "The genomic sequencing analysis of a non-uniformly sequenced genome tends to contain false positive CNA calls as it is computationally challenging to distinguish between amplification bias (i.e., noise) and CNAs (i.e., true signal) for read count fluctuation [41]."

=>

"The analysis of a non-uniformly sequenced genome tends to contain false positive CNA calls as it is computationally challenging to distinguish whether read count fluctuations are due to amplification biases or to true CNAs [41].

3, 13: "Therefore it is important to measure the non-uniformity of genomic coverage before CNA calling." => and how can we measure this? This seems the right place to explain how readers can measure coverage heterogeneity

3, 27: "recovering the lineage of single cells from the same sample." => not sure what you mean by the lineage of single cells, perhaps "a more complete cell genealogy" or, directly, "tumor history"?

3, 39: "coverage fluctuation is not as promising" => What does "is not as promising" means here? I guess you mean larger.

3, 42: "But such improvement is contingent upon prior knowledge of the ITH in the samples, which is unknown and is an objective of the biological studies." => ""But such improvement is contingent upon prior knowledge of the ITH in the cell population, which is rather one of the objectives of the scDNAseq studies"

3, 50: "However, since sequencing is targeted at a catalogue of the designed panel or a custom panel, most of the genome is not covered and the analysis may miss major CNAs by this platform." => "However, since this technology is targeted at small set of genomic regions, major CNAs can be missed"

3, 54: "In addition to low and uneven coverage, scDNAseq data also suffers from a high allelic dropout (ADO) rate, doublets, and a high C>T transition bias. The C>T bias, though being a major confounding issue in SNV calling for causing false positives,"

=> "In addition to low and uneven coverage, scDNAseq data also suffers from a high allelic dropout (ADO) rate, doublets (DEFINE doublets), and a high rate of false C>T calls (add REFERENCES). The C>T bias, though being a major confounding issue in SNV calling,"

3, 58: "The same applies to ADO, as most of the existing CNA calling methods do not distinguish the major and minor alleles." => "The same applies to ADO, as most of the existing CNA calling methods do not distinguish major and minor alleles (DEFINE major and minor alleles)."

4, 12: "and thus violating the assumption of most of the CNA calling methods." => "and thus, violating an assumption of most CNA calling methods."

4, 14: "but the consequence of doublets to breakpoint detection is not as bad as calling the absolute copy numbers" => not sure of what you mean.

4, 15: Cell contamination should be much worse than doublets, as contaminant cells are not, by definition, from the same sample

5, 9: "high false-positive deletion calls" => remove "high" or rephrase using "rate"

5, 36: " For scDNAseq, this could lead to false-negative CNAs, which are hundreds of thousands of base pairs long, as the bin size is often around 200kbp [24]." => remove first comma?

5, 40: "to the dropping read coverage" => "to the dropping OF read coverage"

6, 24: "However, correcting mappability based on a pre-computed mappability file constrains the tool to certain human genome reference versions." => which tool?

6, 35: "The selection process is more flexible and adaptable to the data if it is data-driven." => you mean if it takes into account specific properties of the data at hand?

6, 36: "Ginkgo [51], for example, blacklists "bad bins" using 54 existing normal cells without input from the data." => Ginkgo [51], for example, blacklists "bad bins" using 54 existing normal cells without input from the data AT HAND."

6, 46: "based on the empirically derived distribution." = > "based on this distribution."

6, 51: "Cells with extremely high Gini coefficient are expected to have a large number of false positive CNA calls, and thus shall not be considered in CNA inference." => false positives or false negatives? or both?

7, 28: "…across genomes." => "…across sampled cells"?

7, 61: "…they handle single sample/cell at a time or multiple ones" => "…they handle a single sample/cell at a time, or multiple ones at once."

7, 62: "as the latter potentially fits scDNAseq data better." => exactly why?

8, 24: "a simulation study." => of single cells, correct?

8, 25: "For 1000 cells, it takes about 7 hours to finish…" => and which was the depth?

8, 36: "size and reference version are restricted" => you mean fixed?

8, 44: "It then uses a circular binary segmentation (CBS) [64] based approach for segmentation" = > "It then uses a circular binary segmentation (CBS) [64] approach"

8, 39: Ginkgo can be also run locally…

10, 21: "However, as we discussed, scDNAseq suffers from high ADO, and when alleles are separately taken into account, modeling ADO is essential for achieving high accuracy in segmenting the genome and cell clustering." => Can you justify this statement with data? Or is it a prediction?

10, 39: "This separation of the two processes may propagate errors made in breakpoint identification to the following analysis." => "… to the phylogenetic analysis"

10, 55: "Such design favors large CNAs, as it has a better chance to be identified prior to smaller CNAs." => "Such design favors large CNAs, as they have a better chance to be identified than smaller CNAs."

11, 7: CNA rate variation per se does not need to result in more overlap. Large CNA rates do result in more overlaps, but a large rate is not the same as variable rate. Average (number of events and size), not variance, is what it matters for the overlap issue.

11, 55: You have not explained yet what a "finite-site" model is.

12, 39: "While this level of resolutions" => "While this level of resolution"

12, 51: "Existing Relevant Approaches to Evolutionary Analysis of CNAs" => "Existing Approaches Relevant to the Evolutionary Analysis of CNAs"

12, 54: "In particular, three general approaches from the fields of phylogenetics and molecular evolution and population genetics can be useful, and some readily available, in evolutionary analyses of CNAs or, more generally, copy number profiles." => Several words seem to be missing.

12, 59: "To the best of our understanding, partitioning a genome into bins as described above, and conducting bin-based analyses are most common in this area" => which area?

13, 14: "Assuming the bins are independent makes the analyses more tractable and lend themselves to analyses using existing tools." => analyses lend themselves to analyses? I guess you mean "Assuming the bins are independent makes the analyses more tractable and facilitates the use of existing tools."

13, 47: "To address this problem, distance correction can be applied; " => "To address this problem, a distance correction can be applied; "

14, 13: "An alternative evolutionary model is the stepwise mutation model (SMM). In this model, a mutation can only increase or decrease the state (number of repeats) by 1." => not necessarily by 1, it can be any number, the key is that the states are ordered.

14, 16: "Treating the different copy numbers at a locus similarly to the states of a microsatellite, the infinite-alleles model and SMM, along with methods for inference under them can be applicable to evolutionary analysis of copy number profiles." => to some evolutionary analyses. Phylogenetic trees are seldom built using microsatellites precisely because the models are too naïve.

14, 41: "...or for inferring evolutionary histories of species given a set of multigene families (e.g., [91]) could potentially be applicable to CNA evolutionary modeling and analysis."=> and what would be the "species" in cell populations? In cancer, I would say only samples from different organs would make sense.

15, 13: "elucidating ITH, etc.," => remove "etc.", a reader should not guess what you mean

15, 13 "CNAa" => "CNAs"

15, 47": "very small clones" => you mean "rare" or "infrequent".

Table 1: I would use "Depth" instead of "Coverage".

Table 2: this table is very difficult to read because of uninformative column labels (S,D,1,2,etc.). I suggest you transpose it and use meaningful abbreviations instead of single letters and numbers.

Best wishes,

David Posada

**Authors Response**

**Point-by-point responses to the reviewers’ comments:**

Reviewer #1: page 3 line 54: the degree of these artifacts can depend on the sequencing technology

*We added this note in the manuscript.*

page 15, line 51: SCICoNE does not have an independent bin model, in fact this is their rationale for using an event tree model rather than a cell-based phylogeny. Events are formed of contiguous bins in their model.

*Thanks for pointing this out. We edited the text accordingly.*

The manuscript has several copy-editing errors and would benefit from some proofreading

*We proofread the manuscript and corrected the copy-editing errors.*

Reviewer #3: The authors have made a nice effort to revise the manuscript. Clearly, the scope of the article and the writing style have improved, but I have to say that the level of ambiguity in the text is still significant (see below minor comments).

The section of scDNAseq technologies is an improvement. Table 1 is the type of summary I appreciate in a review. Sections like "General Considerations When Using scDNAseq CNA Detection Methods" also make the review more useful for the reader.

** (relatively) Major comments **

1) Guideline

While now we can see more recommendations regarding experimental design, data analysis, and interpretation. I still miss a condensed guideline for the user to choose the best scCNA caller for the question at hand. Normally such a guideline is the most useful part of a review.

*We added a sentence recommending the best scCNA caller(s) at the end of the subsection “General Considerations When Using scDNAseq CNA Detection Methods”.*

2) Extended concepts

For example, how does the reader exactly measure coverage heterogeneity? In particular, I miss a more detailed explanation of "phase", and allele-specific CNA calls, which indeed they are fundamental for improved CNA evolutionary models.

*We explained how to measure coverage heterogeneity by giving examples at the place where we mentioned the importance of measuring the non-uniformity of genomic coverage before CNA calling.*

*We explained in more detail the allele-specific CNA detection, and their impact on the inference of the evolution from CNA. We also explained “phasing”, and its relationship with scDNAseq in the section “Particularities of scDNAseq”.*

3) Relevant preprint

A very relevant preprint that should be discussed in depth is "Efficient Bayesian inference of phylogenetic trees from large scale, low-depth genome-wide single-cell data" by Dorri et al.; bioRxiv preprint doi: https://doi.org/10.1101/2020.05.06.058180.

*We thank the reviewer for bringing this to our attention. We have addressed this point and added the reference.*

4) Running times

Running times are now included for some, but not all, methods. I still think that it would be very informative for the reader to see the results of the analysis of at least one real data set with the different callers and compare the CN calls, including runtime.

*We are very willing to run all different CNA callers for scDNAseq on a real data set. But we refrain from doing so due to the scope of this paper, which is a review paper instead of a benchmark paper. We leave this work to the next benchmark paper.*

5) Publishing strategy

About publishing the review and the benchmark as two different papers side-by-side, I am OK with it.

*Thank you.*

** Minor specific comments **

[page, line]

Abstract: "Results: In this review, we review eight methods that have been developed for detecting copy number aberration (CNA)" => add "in scDNAseq data".

*Added.*

2, 6: "Moreover, quite a few methods that have been applied to detect CNAs from scDNAseq data were "borrowed" from other types of data" => methods cannot be borrowed from data. One can see what you mean but this sounds too informal; this statement needs to be rewritten.

*Yes, we edited it and made it more formal.*

2, 9: "Since DOP-PCR [17] and TnBC [18], the single-cell DNA amplification protocols generating data suitable for CNA detection, are limited in terms of their coverage depth and uniformity, it is important to study the existing methods in terms of how well they can be adapted to scDNAseq data." => some words seem to be missing

*We rewrote this sentence.*

2, 18: "Finally, while SNVs occur to only one nucleotide" => sounds strange to me

*We rewrote it.*

2, 19: define "focal region".

*Done.*

2, 19: "The former is a result of genomic instability due to segregation error during anaphase [20], whereas the latter is due to the gain of selective advantage during tumor progression [21-23]."=> I am not sure if former here refers to SNVs, or to the whole chromosomal events, I assume it is the latter. Most importantly, that CNAs "are due" to the gain a of a selective advantage does not make sense to me. Selection could never be the "cause" of a change. Moreover, a CNA can become abundant and even clonal just by chance (i.e., neutral mutations can fix in populations with more or less probability depending on multiple factors). As written, it seems that all CNAs we see in a tumor offer selective advantages, which is clearly not the case.

*Thank you for pointing this out. We rewrote the text for clarification.*

2, 59: "far from enough" could be understood as too little or as too much. It is easy to be precise by writing "not enough"

*Edited.*

3, 7: remove "etc.", if you know more methods, enumerate them. Alternatively, if you just want to highlight some examples, use "e.g.".

*Edited.*

3, 10: "The genomic sequencing analysis of a non-uniformly sequenced genome tends to contain false positive CNA calls as it is computationally challenging to distinguish between amplification bias (i.e., noise) and CNAs (i.e., true signal) for read count fluctuation [41]."

=>

"The analysis of a non-uniformly sequenced genome tends to contain false positive CNA calls as it is computationally challenging to distinguish whether read count fluctuations are due to amplification biases or to true CNAs [41].

*Done.*

3, 13: "Therefore it is important to measure the non-uniformity of genomic coverage before CNA calling." => and how can we measure this? This seems the right place to explain how readers can measure coverage heterogeneity

*We added a couple of examples on how to measure the non-uniformity of genomic coverage.*

3, 27: "recovering the lineage of single cells from the same sample." => not sure what you mean by the lineage of single cells, perhaps "a more complete cell genealogy" or, directly, "tumor history"?

*Yes, we meant tumor history. We clarified it.*

3, 39: "coverage fluctuation is not as promising" => What does "is not as promising" means here? I guess you mean larger.

*Yes, we meant that the coverage fluctuation is actually larger. We clarified it.*

3, 42: "But such improvement is contingent upon prior knowledge of the ITH in the samples, which is unknown and is an objective of the biological studies." => ""But such improvement is contingent upon prior knowledge of the ITH in the cell population, which is rather one of the objectives of the scDNAseq studies"

*Done.*

3, 50: "However, since sequencing is targeted at a catalogue of the designed panel or a custom panel, most of the genome is not covered and the analysis may miss major CNAs by this platform." => "However, since this technology is targeted at small set of genomic regions, major CNAs can be missed"

*Done*

3, 54: "In addition to low and uneven coverage, scDNAseq data also suffers from a high allelic dropout (ADO) rate, doublets, and a high C>T transition bias. The C>T bias, though being a major confounding issue in SNV calling for causing false positives,"

=> "In addition to low and uneven coverage, scDNAseq data also suffers from a high allelic dropout (ADO) rate, doublets (DEFINE doublets), and a high rate of false C>T calls (add REFERENCES). The C>T bias, though being a major confounding issue in SNV calling,"

*Done. The definition of doublets and the reference for high rate of false calls were added.*

3, 58: "The same applies to ADO, as most of the existing CNA calling methods do not distinguish the major and minor alleles." => "The same applies to ADO, as most of the existing CNA calling methods do not distinguish major and minor alleles (DEFINE major and minor alleles)."

*Done. The definition of major and minor alleles was added.*

4, 12: "and thus violating the assumption of most of the CNA calling methods." => "and thus, violating an assumption of most CNA calling methods."

*Edited*

4, 14: "but the consequence of doublets to breakpoint detection is not as bad as calling the absolute copy numbers" => not sure of what you mean.

*We meant that the effect of doublets on detection of breakpoints is less than its effect on the inference of absolute copy numbers. We rewrote this sentence.*

4, 15: Cell contamination should be much worse than doublets, as contaminant cells are not, by definition, from the same sample

*Edited: to clarify, we added to that sentence “even to a larger extent as the contaminant cells are not from the sample”*

5, 9: "high false-positive deletion calls" => remove "high" or rephrase using "rate"

*Edited.*

5, 36: " For scDNAseq, this could lead to false-negative CNAs, which are hundreds of thousands of base pairs long, as the bin size is often around 200kbp [24]." => remove first comma?

*Edited.*

5, 40: "to the dropping read coverage" => "to the dropping OF read coverage"

*Done.*

6, 24: "However, correcting mappability based on a pre-computed mappability file constrains the tool to certain human genome reference versions." => which tool?

*We meant “the CNA detection tool”. The text is edited.*

6, 35: "The selection process is more flexible and adaptable to the data if it is data-driven." => you mean if it takes into account specific properties of the data at hand?

*We meant that the threshold that determines which bins are outliers is a function of the data given instead of the hard-coded parameters. We clarified this in the manuscript.*

6, 36: "Ginkgo [51], for example, blacklists "bad bins" using 54 existing normal cells without input from the data." => Ginkgo [51], for example, blacklists "bad bins" using 54 existing normal cells without input from the data AT HAND."

*Edited.*

6, 46: "based on the empirically derived distribution." = > "based on this distribution."

*Done.*

6, 51: "Cells with extremely high Gini coefficient are expected to have a large number of false positive CNA calls, and thus shall not be considered in CNA inference." => false positives or false negatives? or both?

*It is mainly the false positives. The reason is the high Gini coefficient reflects a high fluctuation of the read coverage, which makes the read count noisy and the CNA detection tools may falsely count them as the signals of CNAs.*

7, 28: "…across genomes." => "…across sampled cells"?

*Yes, we rewrote it as “across sampled cells”.*

7, 61: "…they handle single sample/cell at a time or multiple ones" => "…they handle a single sample/cell at a time, or multiple ones at once."

*Done.*

7, 62: "as the latter potentially fits scDNAseq data better." => exactly why?

*We added: “because it pools the information shared among the single cells”.*

8, 24: "a simulation study." => of single cells, correct?

*That is correct. We clarified by adding “of single cells”.*

8, 25: "For 1000 cells, it takes about 7 hours to finish…" => and which was the depth?

*It is 0.04X. We added it to the text. It is also mentioned in the reference.*

8, 36: "size and reference version are restricted" => you mean fixed?

*We meant that there are only a few versions that are available and they are restricted to only a small pool. We clarified it in the text.*

8, 44: "It then uses a circular binary segmentation (CBS) [64] based approach for segmentation" = > "It then uses a circular binary segmentation (CBS) [64] approach"

*Done.*

8, 39: Ginkgo can be also run locally…

*We added the clarification.*

10, 21: "However, as we discussed, scDNAseq suffers from high ADO, and when alleles are separately taken into account, modeling ADO is essential for achieving high accuracy in segmenting the genome and cell clustering." => Can you justify this statement with data? Or is it a prediction?

*This is a prediction based on the signals that CHISEL used and the nature of scDNAseq technologies. The original text explained the reasoning.*

10, 39: "This separation of the two processes may propagate errors made in breakpoint identification to the following analysis." => "… to the phylogenetic analysis"

*Edited.*

10, 55: "Such design favors large CNAs, as it has a better chance to be identified prior to smaller CNAs." => "Such design favors large CNAs, as they have a better chance to be identified than smaller CNAs."

*Edited.*

11, 7: CNA rate variation per se does not need to result in more overlap. Large CNA rates do result in more overlaps, but a large rate is not the same as variable rate. Average (number of events and size), not variance, is what it matters for the overlap issue.

*We meant CNA rates, as we stated in the text, not the CNA rate’s variation. We removed the phrase “especially when coupled with variability in CNA sizes” as we agree with the reviewer that the variability of the CNA size does not lead to more overlapping CNAs.*

11, 55: You have not explained yet what a "finite-site" model is.

*We added the definition in its first occurrence.*

12, 39: "While this level of resolutions" => "While this level of resolution"

*Edited.*

12, 51: "Existing Relevant Approaches to Evolutionary Analysis of CNAs" => "Existing Approaches Relevant to the Evolutionary Analysis of CNAs"

*Edited.*

12, 54: "In particular, three general approaches from the fields of phylogenetics and molecular evolution and population genetics can be useful, and some readily available, in evolutionary analyses of CNAs or, more generally, copy number profiles." => Several words seem to be missing.

*Edited.*

12, 59: "To the best of our understanding, partitioning a genome into bins as described above, and conducting bin-based analyses are most common in this area" => which area?

*“This area” refers to CNA detection. The sentence was edited.*

13, 14: "Assuming the bins are independent makes the analyses more tractable and lend themselves to analyses using existing tools." => analyses lend themselves to analyses? I guess you mean "Assuming the bins are independent makes the analyses more tractable and facilitates the use of existing tools."

*Edited.*

13, 47: "To address this problem, distance correction can be applied; " => "To address this problem, a distance correction can be applied; "

*Done.*

14, 13: "An alternative evolutionary model is the stepwise mutation model (SMM). In this model, a mutation can only increase or decrease the state (number of repeats) by 1." => not necessarily by 1, it can be any number, the key is that the states are ordered.

*Thank you. We edited and clarified this.*

14, 16: "Treating the different copy numbers at a locus similarly to the states of a microsatellite, the infinite-alleles model and SMM, along with methods for inference under them can be applicable to evolutionary analysis of copy number profiles." => to some evolutionary analyses. Phylogenetic trees are seldom built using microsatellites precisely because the models are too naïve.

*Yes, we changed it to “some evolutionary analyses”.*

14, 41: "...or for inferring evolutionary histories of species given a set of multigene families (e.g., [91]) could potentially be applicable to CNA evolutionary modeling and analysis."=> and what would be the "species" in cell populations? In cancer, I would say only samples from different organs would make sense.

*We clarified this by explaining the word “species” in the context.*

15, 13: "elucidating ITH, etc.," => remove "etc.", a reader should not guess what you mean

*Done.*

15, 13 "CNAa" => "CNAs"

*Corrected.*

15, 47": "very small clones" => you mean "rare" or "infrequent".

*Edited.*

Table 1: I would use "Depth" instead of "Coverage".

*Edited.*

Table 2: this table is very difficult to read because of uninformative column labels (S,D,1,2,etc.). I suggest you transpose it and use meaningful abbreviations instead of single letters and numbers.

*This is a huge table. The current design facilitates putting all the information regarding each tool in one row. We doubt we could do this if we don’t use the abbreviations of the single letters and numbers. In addition, the numbers are straightforward as they are the index of the seven steps of the CNA calling. Readers can refer to the table caption for the details of each abbreviation.*

Best wishes,

David Posada
